# Complete chloroplast genome of *Camellia japonica* genome structures, comparative and phylogenetic analysis

**DOI:** 10.1371/journal.pone.0216645

**Published:** 2019-05-09

**Authors:** Wei Li, Cuiping Zhang, Xiao Guo, Qinghua Liu, Kuiling Wang

**Affiliations:** College of Landscape Architecture and Forestry, Qingdao Agricultural University, Qingdao, China; National Cheng Kung University, TAIWAN

## Abstract

*Camellia* is an economically, ecologically and phylogenetically valuable genus in the family Theaceae. The frequent interspecific hybridization and polyploidization makes this genus phylogenetically and taxonomically under controversial and require detailed investigation. Chloroplast (cp) genome sequences have been used for cpDNA marker development and genetic diversity evaluation. Our research newly sequenced the chloroplast genome of *Camellia japonica* using Illumina HiSeq X Ten platform, and retrieved five other chloroplast genomes of *Camellia* previously published for comparative analyses, thereby shedding lights on a deeper understanding of the applicability of chloroplast information. The chloroplast genome sizes ranged in length from 156,607 to 157,166 bp, and their gene structure resembled those of other higher plants. There were four categories of SSRs detected in six *Camellia* cpDNA sequences, with the lengths ranging from 10 to 17bp. The *Camellia* species exhibited different evolutionary routes that lhbA and orf188, followed by orf42 and psbZ, were readily lost during evolution. Obvious codon preferences were also shown in almost all protein-coding cpDNA and amino acid sequences. Selection pressure analysis revealed the influence of different environmental pressures on different *Camellia* chloroplast genomes during long-term evolution. All *Camellia* species, except *C*. *crapnelliana*, presented the identical rate of amplification in the IR region. The datasets obtained from the chloroplast genomes are highly supportive in inferring the phylogenetic relationships of the *Camellia* taxa, indicating that chloroplast genome can be used for classifying interspecific relationships in this genus.

## Introduction

*Camellia*, containing about 280 species, is a genus with high economic, ecological and phylogenetic values in the family Theaceae [1, 2]. It is native to Southern, Eastern Asia and China, which possess more than 80% of the species and are the center of species diversity [3]. Besides the abundance in species diversity and phylogenetic significance, people pay more attention to this genus, for their commercial and ornamental values. For example, *C*. *sinensis var*. *sinensis* and *C*. *sinensis var*. *assamica* have the highest economic value in *Camellia*. Tea leaves have been proven to be beneficial for human health as they contain over 700 chemical constituents [1, 4]. *Camellia* is also known as ornamental trees for urban gardening. The cultivation history of *Camellia* has been at least 1300 years in China [3]. Today, a group of yellow flowers named golden *Camellia*, e.g. *C*. *chrysantha*, are grown for ornamental purposes, with thirteen to sixteen petals of a flower and blooming several times in a year. Many other *Camellia* species, e.g. *C*. *japonica*, also had local uses. The other most economically valuable species, *C*. *oleifera* and *C*. *reticulata*, are used for edible oil and cooking in China [5, 6]. At present, more than 3 million hectares are used for *Camellia* oil production, and the yield of the *Camellia* nearly 164,000 tons of edible oil [3, 7]. Although the *Camellia* is native to Asia, because of its variety use, the cultivated species are now found all over the world [1, 8–10]. However, the genus *Camellia* is phylogenetically and taxonomically under controversial that detailed investigation is required, as a result of frequent interspecific hybridization and polyploidization. Whereas classification of the genus *Camellia* is traditionally based on morphology [11–16], the result of this systematics is often unreliable and made lots of controversy as morphology is often affected by environmental factors [2]. As a result, it is urgent to seek other methods for rebuilding the classification of *Camellia*.

Being relatively stable and not easily affected by the environment, molecular methods can provide useful information for taxonomic classification and phylogenetic. Molecular methods, e.g. DNA and RNA sequences [10, 17–24], internal transcribed spacer [10, 18], simple sequence repeats (SSR) [25, 26], ribosomal DNA [27] and several DNA loci [28], have been involved to better understand the evolution of the *Camellia*. A number of studies focus on the taxonomy, species identification and phylogenetics of the *Camellia*, but still have not get a satisfied resolution. A recent study used complete chloroplast genomes in several *Camellia* species [2, 29–31] and got more information of this species. *Camellia* japonica has been present in Qingdao, Shandong province since the tertiary, it has evolved in dependently after that. A recent research shows that the early flower development sequence placed *C*. *japonica* (Naidong) in a most primitive branch of the phylogenetic tree compared to other species [32]. The taxonomy of *C*. *japonica* with other *Camellia* species is in dispute.

In plant cells, the chloroplast is not only the most important and universal organelle, but also one of the major genetic systems (the other two are nucleus and mitochondria). It is involved in photosynthesis and associated with metabolism, such as fatty acid and amino acid synthetic pathways [33–35]. As an independent organelle, the chloroplast has its own genome. It has a covalently closed circular DNA structure and exists in multi-copies in plant cell. It has a conserved circular DNA arrangement [36]. Since the chloroplast genome is self-replicating and has a relatively independent evolutionary process, it has been used for resolving the source populations during species evolution [8, 34, 37–45].

Here, we report newly sequenced complete chloroplast genomes of *C*. *japonica* using next generation sequencing technology and genomic comparative analysis with other five published chloroplast genome sequence download from the NCBI. This study aims to deeply analyze the chloroplast genomes of six *Camellia* species and to determine their (especially the *C*. *japonica*) phylogenetic positions.

## Materials and methods

### Ethics statement

College of Landscape Architecture and Forestry, Qingdao Agricultural University has had a permit from local forestry authorities (Qingdao forestry bureau http://ly.qingdao.gov.cn/) to collect the sample. This research was carried out in compliance with the laws of People’s Republic of China.

### Plant materials and genomic DNA isolation

We collected fresh leaves from an adult *C*. *japonica* tree growing in the Daguan Island (Jimo District Qingdao city, Shandong, China) (N36°14′, E120°46′, Altitude 10m). The leaves were dried immediately with silicagel. Total genomic DNA was extracted according to Wiland-Szymańska [46].

### Chloroplast genome sequencing, assembly and annotation

We used an ultrasonicator to randomly fragment the extracted genomic DNA into 400–600 bp. The NEBNext Ultra DNA Library Prep Kit was used to construct an Illumina paired-end cpDNA library. Paired-end sequencing (2 × 150 bp) was run on an Illumina HiSeq X Ten platform. After filtering the raw data, the cp genomes were assembled according to the following steps. Frist the clean reads were used to assembled into contigs using SOAPdenovo 2.017. Then, the contigs were aligned to the relative species (*C*. *sinensis* JQ975030) and get the relative location of the contig sequences and the structure diagram of cp genomes were obtained. The software Gap Closer 1.12 were used to fill the gaps. Finally, the complete cp genome sequence were obtained. The chloroplast genome sequences were annotated with CpGAVAS software and DOGMA software, and then manually corrected.

### Molecular marker development

We conducted a sliding window analysis and used DnaSP (DNA Sequences Polymorphism version 5.10.01) software to calculate the nucleotide diversity (Pi) of the six complete *Camellia* chloroplast genomes [47]. We set the step size to 200 bp with a window length of 600bp.

For different lengths of SSRs, including mono-, di-, tri-, tetra-, penta-, and hexa-nucleotides, minimum numbers (thresholds) were 10, 5, 4, 3, 3, and 3, respectively. We manually verified all the repeats found, and removed unwanted results.

The mVISTA[48] program was used to compare the complete chloroplast genome of C. *japonica* to other five published chloroplast genomes of the genus *Camellia*, i.e., *C*. *huana* (KY_626042), *C*. *crapnelliana* (KF_753632), *C*. *azalea* (KY_856741), (KY_626042), *C*. *liberofilamenta* (KY_626041) with the shuffle-LAGAN mode [49], so that inter-and intra-specific variations were shown. We used Mega 6.0 software [50] to determine the variable and parsimony-informative base sites across the complete chloroplast genomes, and LSC, SSC and IR regions of the six chloroplast genomes.

DnaSP software was used for manual detection of insertions/deletions. To estimate selection pressures, the 78 protein coding genes in the chloroplast genomes were combined. We used PAML with the yn00 program to calculate the Ka and Ks rates of the combined sequences [51].

### Selection pressure analysis

We used KaKs_Calculator 2.0 [52] to determine the *Ka* and *Ks* values of genes containing SNP variations, so that we can analyze how different *Camellia* have evolved under different environmental pressures. We also analyzed the codon preference, and mapped them by R software.

Phylogenies were constructed using the 19 cp genome of the *Camellia* species sequences from the NCBI Organelle Genome and Nucleotide Resources database: *C*. *crapnelliana* (KF_753632), *C*. *azalea* (KY_856741), *C*. *luteoflora* (KY_626042), *C*. *huana* (KY_626042), *C*. *liberofilamenta* (KY_626041), *C*. *oleifera* (JQ_975031), *C*. *taliensis* (KF_156836), *C*. *yunnanensis* (KF_156838), *C*. *cuspidate* (KF_156833), *C*. *reticulate* (KJ_806278), *C*. *pitardii* (KF_156837), *C*. *danzaiensis* (KF_156837), *C*. *petelotii* (KJ_806276), *C*. *leptophylla* (KJ_806275), *C*. *impressinervis* (KF_156835), *C*. *grandibracteata* (KJ_806274), *C*. *sinensis* (JQ_975030), *C*. *pubicosta* (KJ_806277). We implemented maximum likelihood (ML) analyses on the CIPRES cluster1[53]. GTR+I+R was selected as the nucleotide substitution model. This model was determined from jModel Test v2.1.4 [54]. This model is used to obtain the dataset from the chloroplast genome. For protein-coding regions, it is also used as a partitioned model.

We also used PAUP v4b10 to analyze maximum parsimony (MP). We treated gaps as missing, and character states as unordered. We selected MULPARS option when performing Heuristic search. Further steps include tree bisection-reconnection branch swapping, and random stepwise addition with 1,000 replications.

## Results

### Basic characteristics of the *Camellia* chloroplast

**Fig 1:**
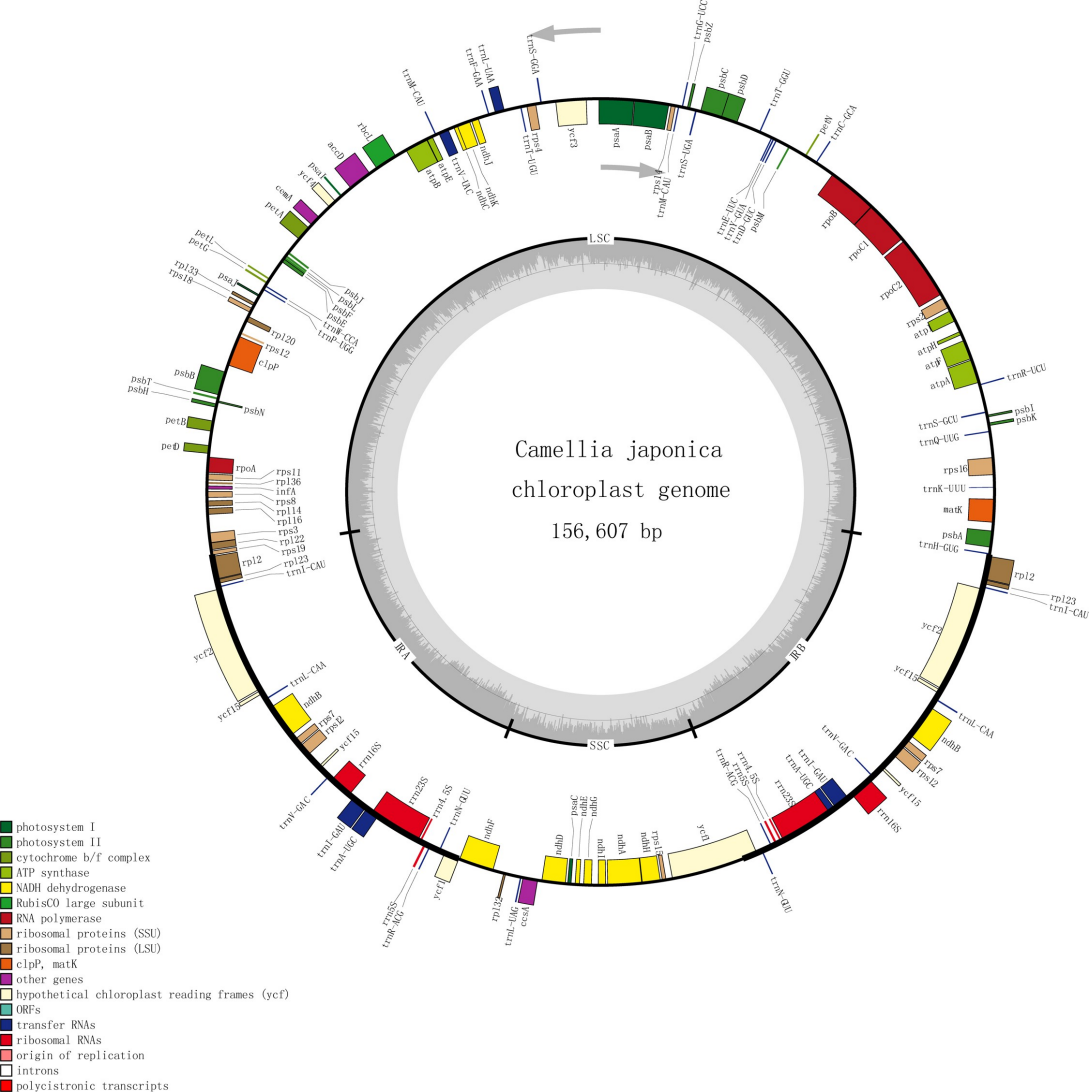
Gene map of *Camellia japonica*. The genes inside and outside of the circle are transcribed in the clockwise and counterclockwise directions, respectively. Genes belonging to different functional groups are shown in different colors. The thick lines indicate the extent of the inverted repeats (IRa and IRb) that separate the genomes into small single-copy (SSC) and large single-copy (LSC) regions.

A total of with 10.44 Gb clean data were generated by Illumina HiSeq X Ten platform. After data filtering with mean Q20 higher than 94.70% and the mean length was 150 bp. The chloroplast genome sizes of the six *Camellia* species ranged from 156,607 bp (*C*. *japonica*) to 157,166 bp (*C*. *luteoflora*). The structure of all chloroplast genomes is quadripartite, which is typical of angiosperm cpDNA. Each chloroplast genome consists of a large single copy region (86,258–86,719bp) and a small single copy region (18,203–18,406bp), separated by two inverted repeat regions (25,967–26,077bp) (Table 1). The GC content of three *Camellia* species (*C*. *japonica*, *C*. *huana and C*. *liberofilamenta*) was 37.32% and the others were 37.30%. The average of GC content is almost similar among the six *Camellia* species. The well-conserved genomic structure resembled those of other higher plants, including gene number and gene order (Fig 1 and Table 2). The complete *C*. *japonica* cp genome sequence has been submitted to GenBank with the accession number PRJNA510919.

**Table 1:** Statistics on the basic features of the chloroplast genomes of six *Camellia* species.

	*C*. *japonica*	*C*. *crapnelliana*	*C*. *azalea*	*C*. *luteoflora*	*C*. *huana*	*C*. *liberofilamenta*
Length (bp)	156607	156997	157039	157166	156903	156865
GC content (%)	37.32	37.30	37.30	37.30	37.32	37.32
AT content (%)	62.68	62.70	62.70	62.70	62.68	62.68
LSC length (bp)	86258	86655	86674	86719	86568	86579
SSC length (bp)	18415	18406	18281	18293	18203	18236
IR length (bp)	25967	25968	26042	26077	26066	26025
Gene number	134	136	135	133	133	133
Gene number in IR regions	36	35	36	35	35	35
Pseudogene number	1	0	3	1	1	1
Pseudogene (%)	0.75	0	2.22	0.75	0.75	0.75
Protein-coding gene number	89	89	87	87	87	87
Protein-coding gene (%)	66.42	65.44	64.44	65.41	65.41	65.41
rRNA gene number	8	8	8	8	8	8
rRNA (%)	5.97	5.88	5.93	6.02	6.02	6.02
tRNA gene number	36	39	37	37	37	37
tRNA (%)	26.87	28.68	27.41	27.82	27.82	27.82

**Table 2:** Genes identified in the chloroplast genome of *Camellia* species.

Category for genes	Group of gene	Name of gene
Genes for photosynthesis	ATP synthase	atpA,atpF(pseudogene),atpH,atpI,atpE,atpB
	NADH-dehydrogenase	ndhJ,ndhK,ndhC,ndhB,ndhF,ndhD,ndhE,ndhG,ndhI,ndhA,ndhH,ndhB
	cytochrome b/f complex	petN,petA,petL,petG,petB,petD
	photosystem I	psaB,psaA,psaI,psaJ,psaC
	photosystem II	psbA,psbK,psbI,psbM,psbD,psbC,psbZ,psbJ,psbL,psbF,psbE,psbB,psbT,psbN,psbH
	Rubisco	rbcL
Transcription and translation related genes	transcription	rpoC2,rpoC1,rpoB,rpoA
	ribosomal proteins	rps12, rps16, rps2, rps14, rps4, rps18, rps12, rps11, rps8, rps3, rps19, rps7,rps15,rps7,rpl33,rpl20,rpl36,rpl14,rpl16,rpl22,rpl2,rpl23,rpl32,rpl23,rpl2
RNA genes	ribosomal RNA	rrn16S,rrn23S,rrn4.5S,rrn5S,rrn5S,rrn4.5S,rrn23S,rrn16S
	transfer RNA	trnH-GUG, trnK-UUU,trnQ-UUG,trnS-GCU,trnR-UCU,trnC-GCA,trnD-GUC,trnY-GUA, trnE-UUC,trnT-GGU,trnS-UGA,trnG-UCC,trnM-CAU,trnS-GGA,trnT-UGU,trnL-UAA, trnF-GAA, trnV-UAC,trnM-CAU,trnW-CCA,trnP-UGG,trnI-CAU,trnL-CAA,trnV-GAC, trnI-GAU, trnA-UGC,trnR-ACG,trnN-GUU,trnL-UAG,trnN-GUU,trnR-ACG,trnA-UGC, trnI-GAU,trnV-GAC,trnL-CAA,trnI-CAU
Other genes	RNA processing	matK
	carbon metabolism	cemA
	fatty acid synthesis	accD
	proteolysis	clpP
Genes of unkown function	Conserved open reading frames	ycf3,ycf4,ycf2,ycf15,ycf15,ycf1,ycf1,ycf15,ycf15,ycf2

### Comparative analysis of the *Camellia* chloroplast genomes

**Fig 2:**
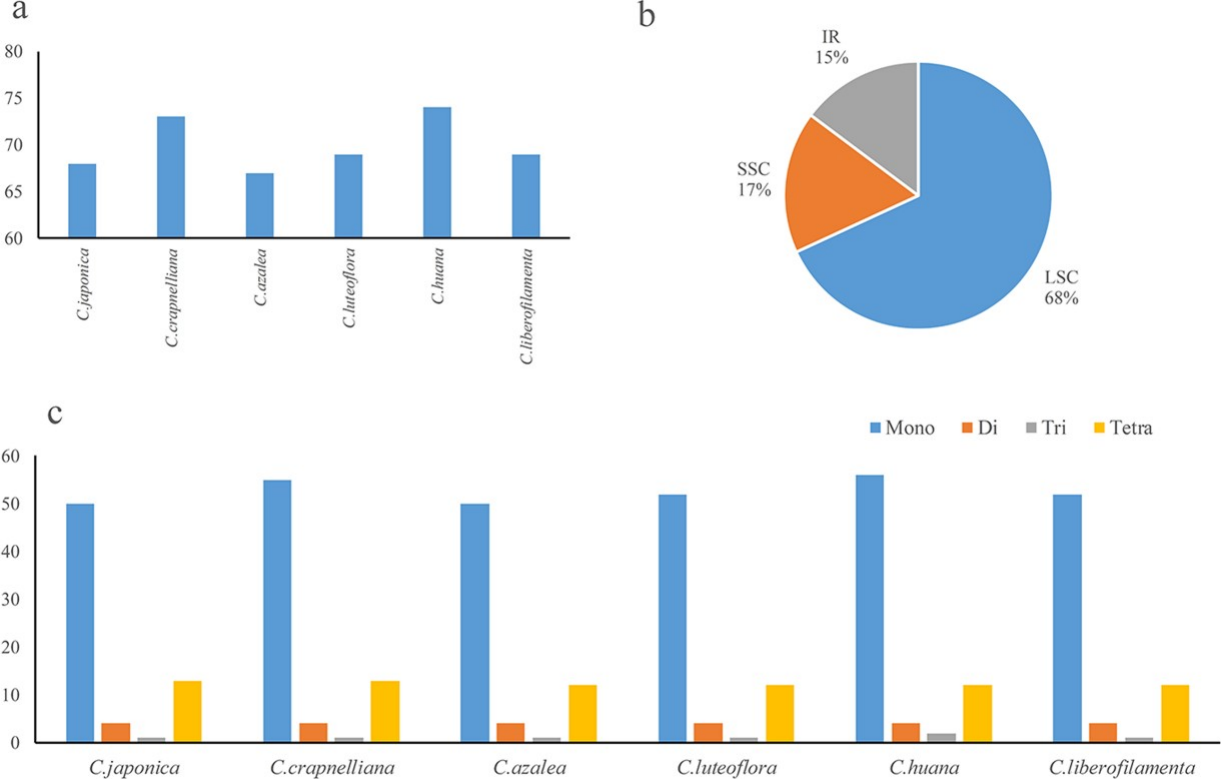
Comparison of simple sequence repeats among six chloroplast genomes. a. Numbers of SSRs detected in ten *Camellia* chloroplast genomes; b. Frequencies of identified SSRs in LSC, IR and SSC regions; c. Numbers of SSR types detected in ten *Camellia* chloroplast genomes.

Chloroplast simple sequence repeats (cpSSRs) play a crucial role in studying phylogeny and population genetics[55]. We analyzed cpSSRs in the chloroplast genomes (S1 and S2 Tables). The number of cpSSRs ranged from 67 (*C*.*azalea*) to 74 (*C*.*huana*) among the six *camellia* taxa. The number of nucleotide repeats had no significant difference among the six *camellia* taxa (Fig 2).

The majority of the 420 SSR loci reside in LSC regions (286 loci, 68.10%). Only a minor portion are located in the SSC regions (72 loci, 17.14%) and IR regions (62 loci, 14.76%). Same as previously reported the SSR loci exhibited a significantly variable distribution among all regions in each of the six *Camellia* chloroplast genomes [34, 56]. The lowest value (17) was between *C*. *huana* and *C*. *luteoflora*, while the highest value (58) of nucleotide substitutions was observed between *C*. *huana* and *C*. *liberofilamenta*, showing a wider range of variability according to the sequence alignment of the six chloroplast genomes (Table 3). The values of *Ka/Ks* ranged from 0.2342 to 0.5971. The lowest value was between *C*. *crapnelliana* and *C*. *luteoflora*. The highest value was between *C*. *huana* and *C*. *liberofilamenta* (Table 3). The result that the *Ka/Ks* ratio is below 1 indicated negative selection as the selection model for the related gene regions.

**Table 3:** Pairwise substitution rate between the *Camellia* chloroplast gemomes based on the 78 protein-coding gene sequences.

	*C*. *japonica*	*C*. *crapnelliana*	*C*. *azalea*	*C*. *luteoflora*	*C*. *huana*	*C*. *liberofilamenta*
*C*. *japonica*		35	33	37	34	33
*C*. *crapnelliana*	0.4200		26	34	58	54
*C*. *azalea*	0.4791	0.3613		20	44	40
*C*. *luteoflora*	0.3872	0.2342	0.3663		17	18
*C*. *huana*	0.4870	0.3610	0.5096	0.4993		23
*C*. *liberofilamenta*	0.4301	0.3470	0.4450	0.4605	0.5971	

### Chloroplast gene gain-loss events

Despite high conservation of chloroplast genome sequences, structural variations, gene loss, and metastasis occur in some species as a result of evolution. This study compared nineteen *Camellia* species (Table 4). We found that lhbA and orf188, followed by orf42 and psbZ, were readily lost during evolution. The results also showed that psaJ, psbF, psbH and psbZ were lost in *C*.*danzaiensis*, compared with the other eighteen species. Moreover, *C*.*japonica* had lost trnfM-CAU gene compared the other species. Gene loss events also occur in other plants, e.g. the loss of infA in the Fagales chloroplast genome [55], and the loss of rpl32 in the *Paeonia obovata* chloroplast genome [57]. Among the rpsl6, ndh, infA, and ycf2 genes, some have disappeared in some angiosperms, and in some legumes, gene loss events have occurred to all of them [58].

**Table 4:** Genes from the chloroplast genomes of *Camellia*.

Name of species	lhbA	orf188	orf42	psaJ	psbF	psbH	psbZ	trnfM-CAU
*C*.*japonica*	-	-	-	+	+	+	+	-
*C*.*azalea*	-	-	+	+	+	+	+	+
*C*.*luteoflora*	-	-	-	+	+	+	+	+
*C*.*liberofilamenta*	-	-	-	+	+	+	+	+
*C*.*huana*	-	-	-	+	+	+	+	+
*C*.*reticulate*	-	-	+	+	+	+	+	+
*C*.*pubicosta*	-	-	+	+	+	+	+	+
*C*.*petelotii*	-	-	+	+	+	+	+	+
*C*.*leptophylla*	-	-	+	+	+	+	+	+
*C*.*grandibracteata*	-	-	+	+	+	+	+	+
*C*.*crapnelliana*	-	+	+	+	+	+	+	+
*C*.*yunnanensis*	+	+	+	+	+	+	-	+
*C*.*pitardii*	+	+	+	+	+	+	-	+
*C*.*taliensis*	+	+	+	+	+	+	-	+
*C*.*impressinervis*	+	+	+	+	+	+	-	+
*C*.*danzaiensis*	+	+	+	-	-	-	-	+
*C*.*cuspidate*	+	+	+	+	+	+	-	+
*C*.*oleifera*	-	-	+	+	+	+	+	+
*C*.*sinensis*	-	-	+	+	+	+	+	+
Total number of missing gene	13	12	4	1	1	1	6	1

### Analysis of codon preference

**Fig 3:**
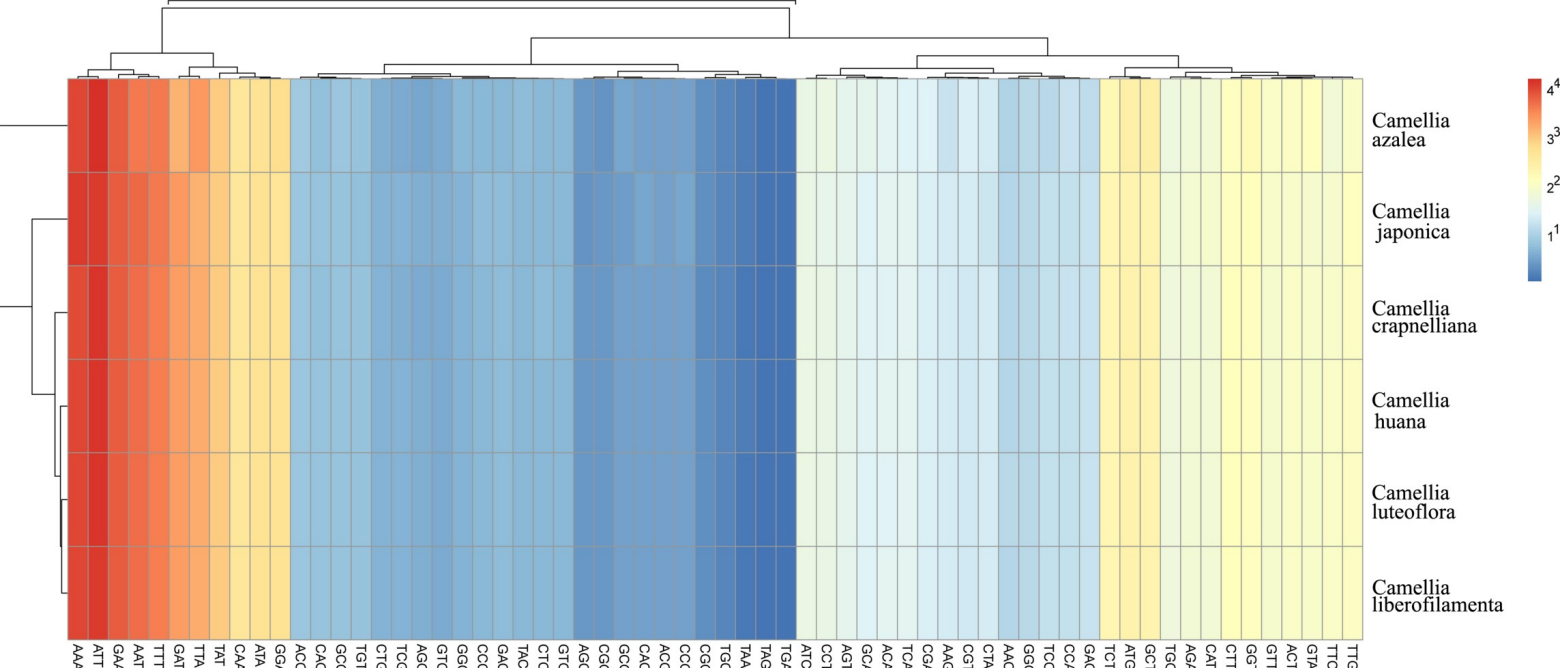
Codon distribution of all merged protein-coding genes. Color key: Red indicates a higher frequency and blue indicates a lower frequency.

69.59% of the *Camellia* chloroplast genome sequence was gene coding, of which the vast majority was protein coding. The analytic varieties provided by statistical analyses of all protein-coding cpDNA and amino acid sequences showed obvious codon preferences. It also showed the similarity of protein codons in the six *Camellia* species, of which AAA, ATT, GAA, AAT, and TTT had the highest frequencies, and the TGA, TAG, TAA, TGC, CGC had the lowest frequencies (Fig 3). The third codon showed a high A/T preference, which is a common phenomenon in higher plant chloroplast genomes [59–61].

The pattern of the codon preference has important significance in studying species evolution. We used the relative synonymous codon usage (RSCU) as a relative intuitionistic to measure the extent of codon bias [62]. The synonymous codon preference is partitioned into four models: high preference (RSCU>1.3), moderate preference (1.2≤RSCU≤1.3), low preference (1.0<RSCU<1.2) and no preference (RSCU≤1.0).

**Fig 4:**
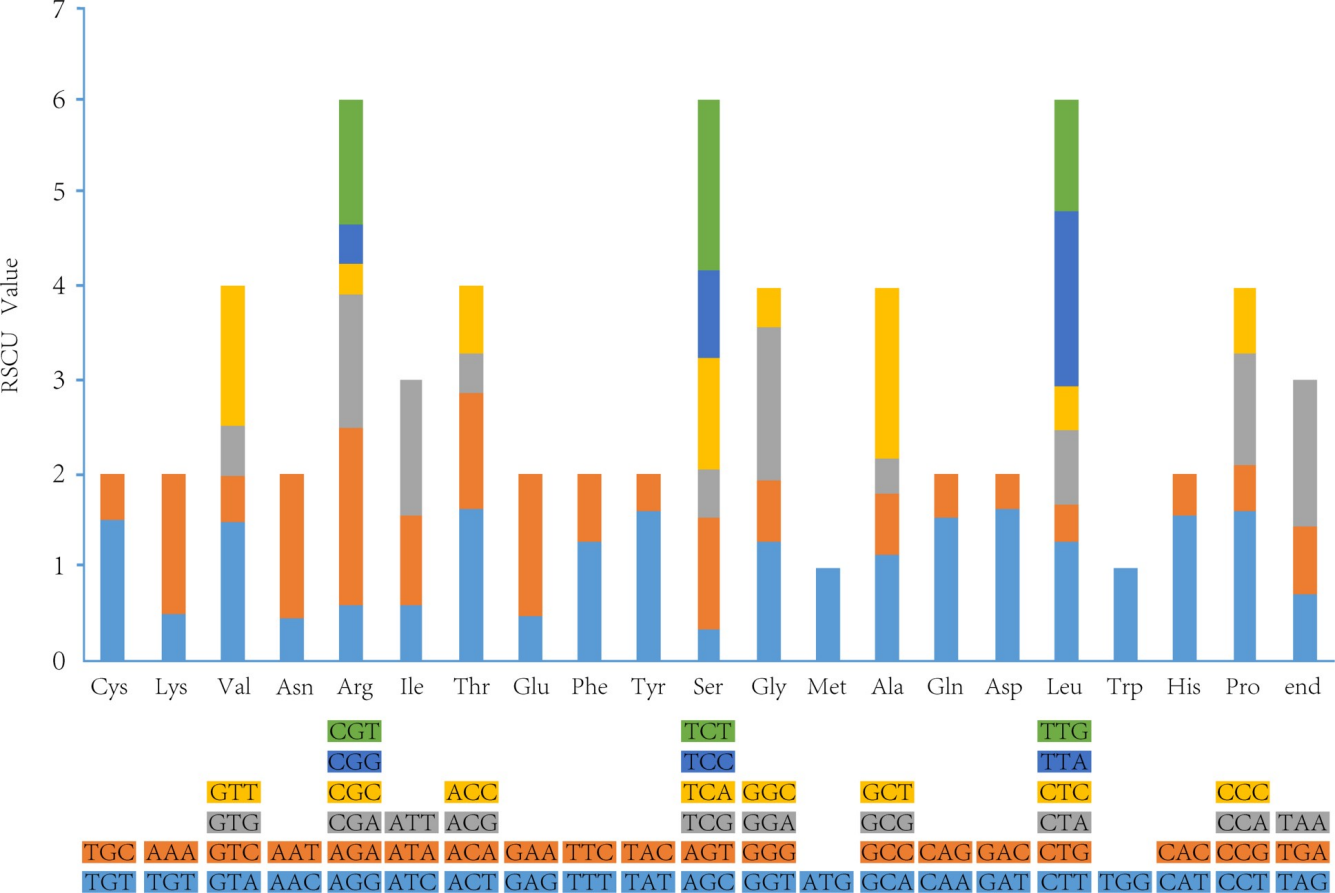
Codon content of 20 amino acid and stop codons in all protein-coding genes.

Among the protein-coding chloroplast genes in the six *Camellia* species, the 20 amino acids were encoded by 64 codons, in which most of the amino acids had codon preferences except tryptophan (Fig 4). As a total, 30 codon preferences were identified, with 18 amino acids and one stop codon involved. Among the preferred codons, 70.00% exhibited high preferences. This result further revealed the relative conservation of *Camellia* chloroplast genomes, as high codon preference is also a common phenomenon in higher plants.

### IR contraction analysis

**Fig 5:**
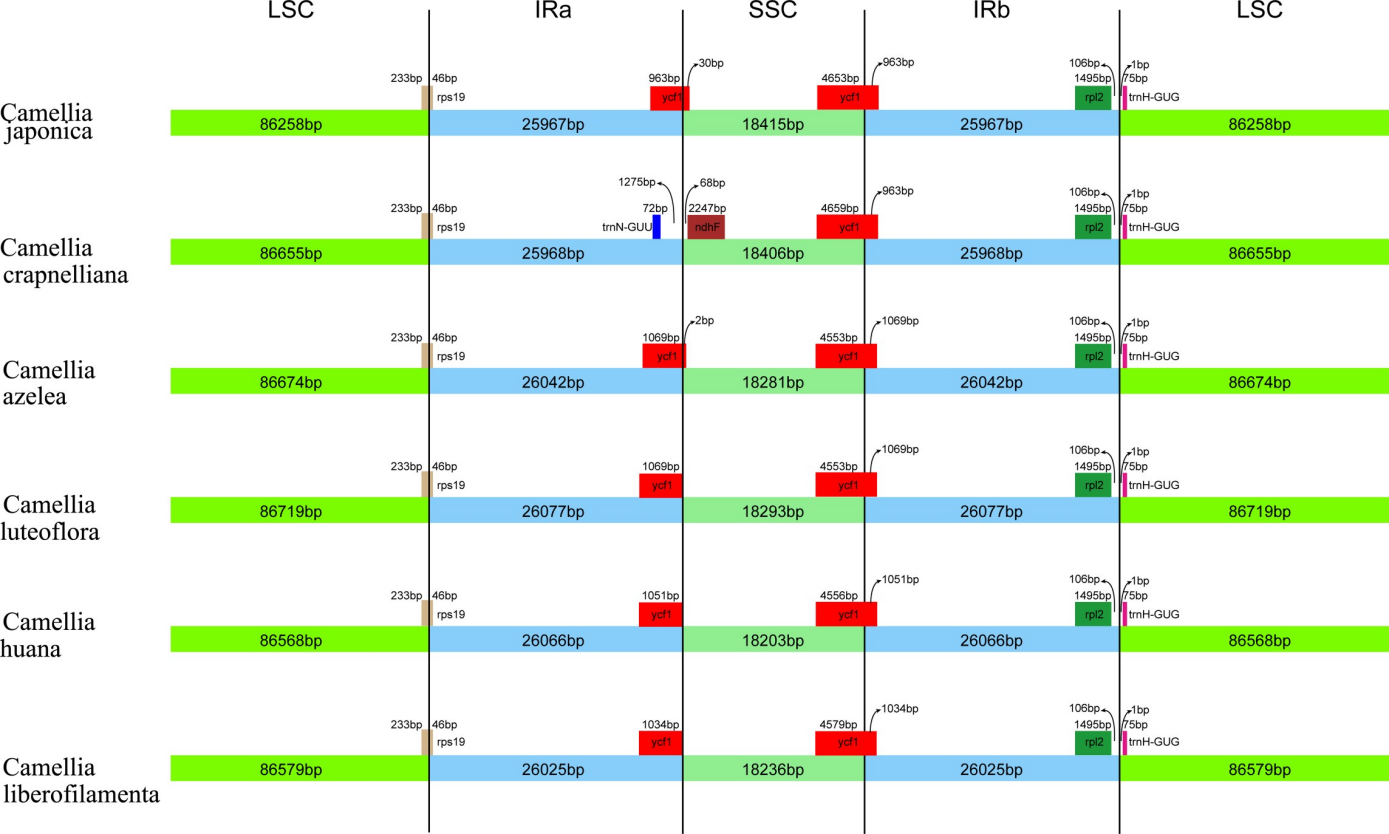
Inverted repeat region contraction analysis of various plant species.

In the chloroplast genome, the IR region is considered as the most conserved region. However, genome size variations often occur in its expansion/contraction regions among various plant lineages, which can be used to study the phylogenetic classification of plants [63]. We compared the IR-SSC and IR-LSC boundaries information of six *Camellia* were compared (Fig 5). The LSC/IRa boundaries was located within the coding region of rps 19 and created a pseudogene of 279bp at LSC/IRa border. The ycf1 gene spanned the IRb/SSC region and the length of ycf1 was from 936bp to 1069bp. Furthermore, the TrnH-GUG gene (75bp) was located in the LSC. However, the gene trn-GUU and ndhf was not observed in *Camellia* except *C*. *crapnelliana*, that means they contribute little to the overall size variations in the chloroplast genomes of *Camellia* plants.

### Genome divergence between the *Camellia* species

**Fig 6:**
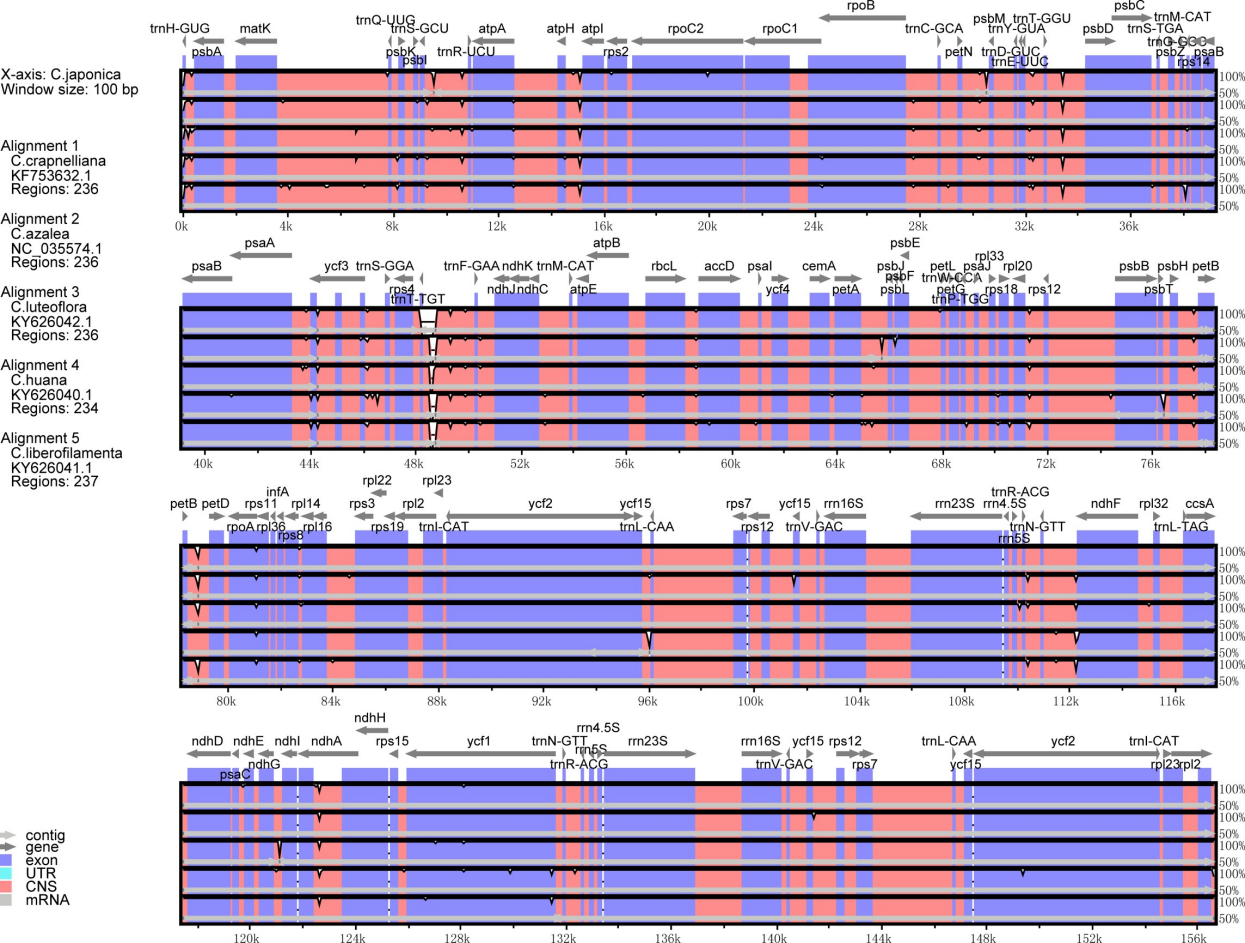
Identity plot comparing the chloroplast genomes of six *Camellia* taxa. The vertical scale indicates the percentage of identity, ranging from 50% to 100%. The horizontal axis indicates the coordinates within the chloroplast genome. Genome regions are color coded as protein-coding, rRNA, tRNA, intron, and conserved non-coding sequences.

A sequence identity analysis based on mVISTA was performed between six *Camellia* species, and the reference was the *C*. *japonica* chloroplast genome (Fig 6). The aligned sequences that exhibit high similarity showed higher conservation than the remaining sequences across the whole chloroplast genome. Lower divergence levels were exhibited in IR and coding regions than in SC and non-coding regions, respectively.

**Fig 7:**
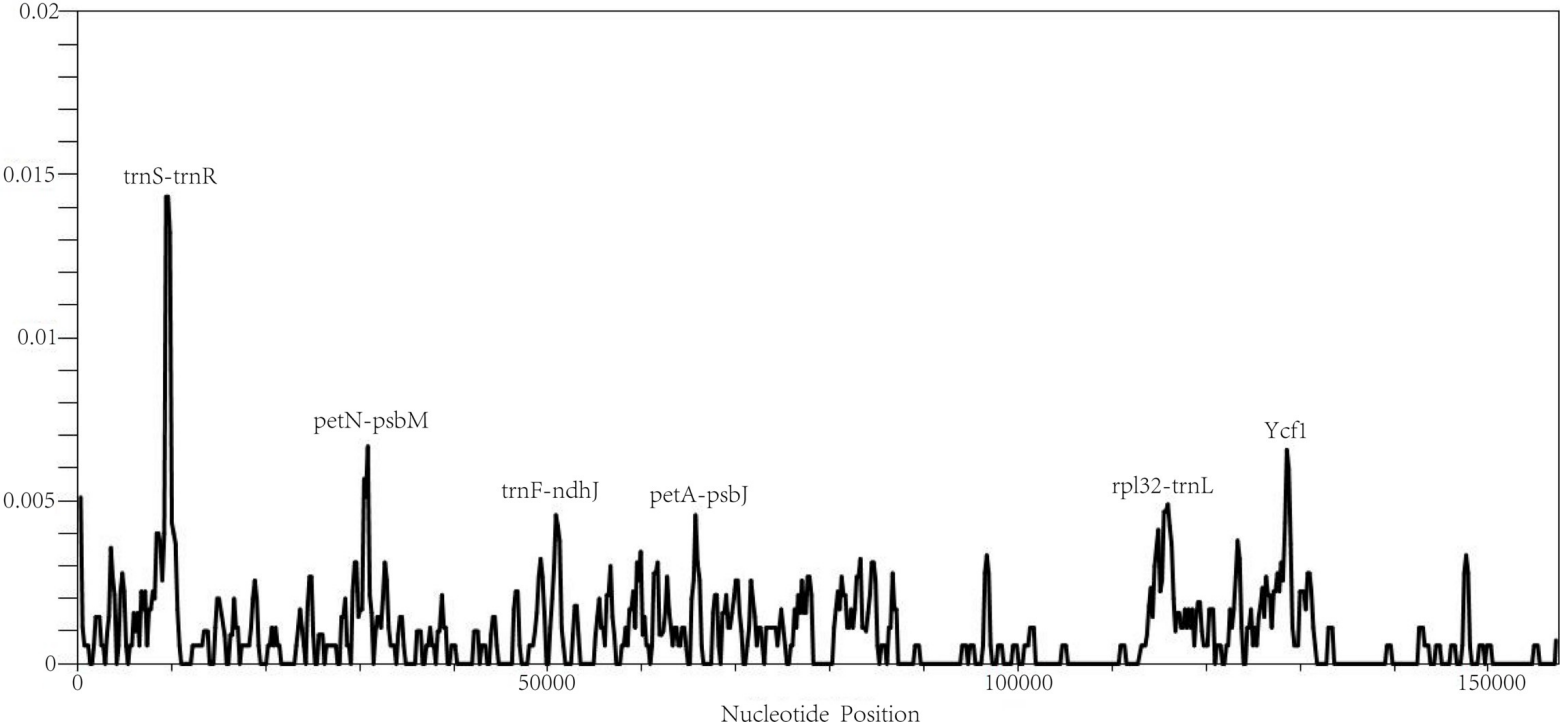
Sliding window analysis of the whole chloroplast genomes of six *Camellia* taxa (window length: 600 bp, step size: 200bp). X-axis, position of the midpoint of a window; Y-axis, nucleotide diversity of each window.

We conducted a sliding window analysis and DnaSP software to calculate the nucleotide diversity of the six complete *Camellia* chloroplast genomes (Fig 7) Among the six *Camellia* taxa, *C*. *japonica* had the most nucleotide substitutions and insertions/deletions, while *C*. *huana* had least nucleotide diversity, and the smallest numbers of nucleotide substitutions and insertions/deletions.

According to the chloroplast genome sequence alignment of the six *Camellia* taxa, six hyper-variable regions, trnS-trnR, petN-psbM, trnF-ndhJ, petA-psbJ, rpl32-trnL, ycf1 were discovered (Fig 7). These six sequences could be used as DNA markers for classification and revealing the genetic divergence of the *Camellia* taxa, with a high discrimination success ranging from 60% to 100% (Table 5). The sequences of the petN-psbM and ycf1 are two most rapidly evolving regions were able to discriminate all the taxa investigated in this study. In those most rapidly evolving regions, 121 and 122 variable base sites were detected, respectively, of which, 61 and 62 informative base sites, made up 2.86–2.99% in each of the sequences. Comparatively, the commonly recommended DNA fragments (rbcL and matK) achieved only 40% and 80% of discrimination success respectively.

**Table 5:** Variability of six hyper-variable markers and universal chloroplast DNA barcodes (rbcL and matK) in *Camellia*.

Maker	length	Variable base sites		Informative base sites		Mean distance	Discriminationsuccess(%) based on Distance method
		Number	Percentage (%)	Number	Percentage (%)		
trnS-trnR	1024	55	5.37	29	2.78	0.0172	60
petN-psbM	2038	121	5.94	61	2.99	0.0157	100
trnF-ndhJ	867	48	5.53	25	2.88	0.0167	80
petA-psbJ	1547	86	5.60	44	2.84	0.0165	80
rpl32-trnL	2017	108	5.35	55	2.72	0.0178	80
ycf1	2168	122	5.63	62	2.86	0.0181	100
rbcL	1401	31	2.22	17	1.18	0.0063	40
matK	1535	49	3.19	26	1.66	0.0091	80

### Phylogenetic analysis

**Fig 8:**
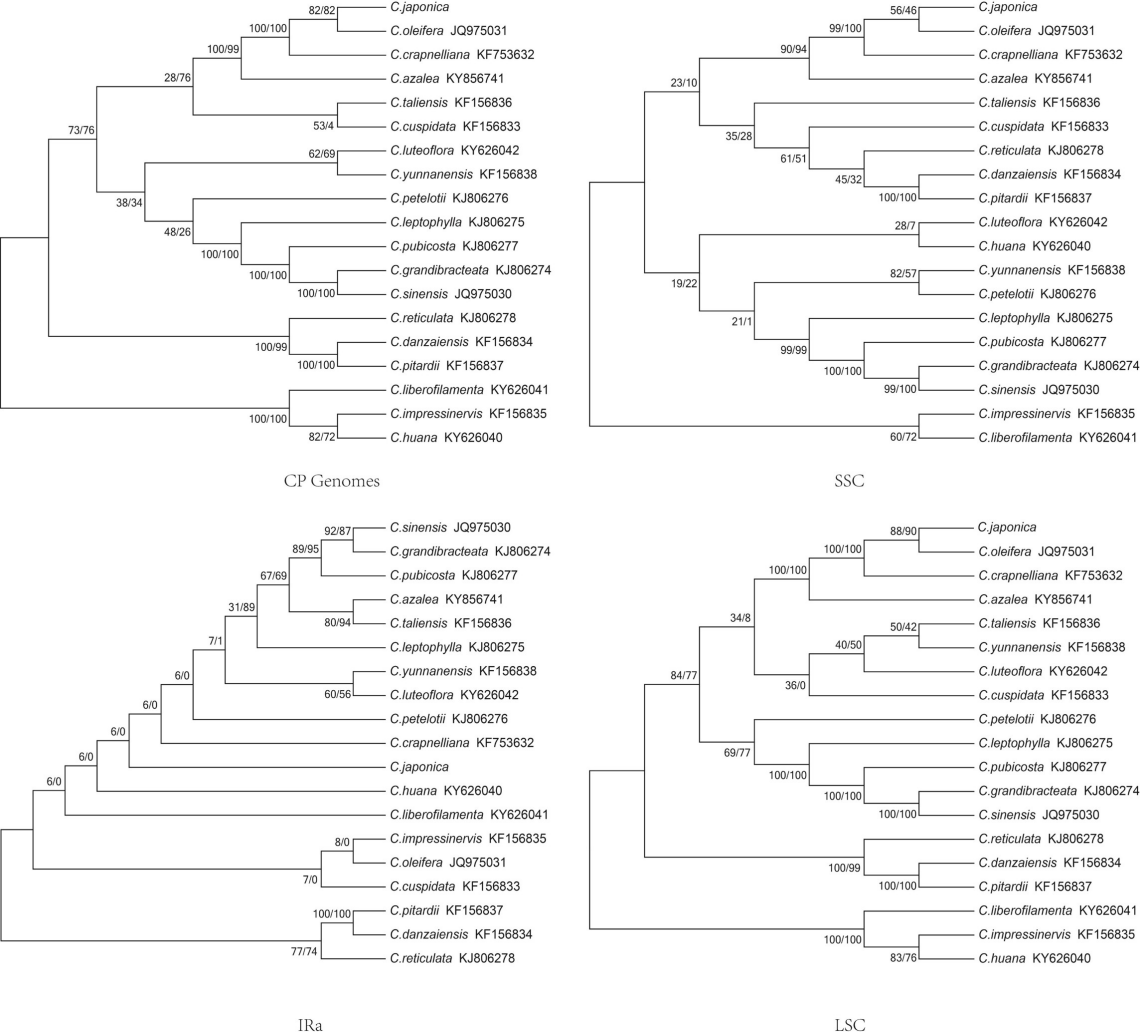
Phylogenetic relationships of the nineteen Camellia species constructed from the complete chloroplast genome sequences using maximum likelihood (ML) and maximum parsimony methods (MP).

Previous studies have fairly well resolved the relationships between *Camellia* species, but have not well studied the position of *Camellia* [29, 30, 64]. Six data partitions including coding regions, large single-copy region, the small single copy region, IR region, the inverted repeat region, introns and spacers and the complete cp DNA sequences from the 19 *Camellia* were used for phylogenetic analyses. All six datasets produced similar phylogenetic trees with moderate to high support, whereas the IR dataset had poor support (Fig 8). The reconstructed phylogeny divided the species into clads based on maximum likelihood (ML) and Maximum parsimony methods (MP).

The phylogenetic tree reveals that *C*. *japonica* is most related with *C*. *oleifera*. Furthermore, the phylogenetic result is consistent with the section-level classification by Raven [65]. The chloroplast resource will be helpful for the conservation, taxonomy, and breeding programs of the genus *Camellia*.

### Chloroplast genome variation and evolution

In this study, Illumina next-generation technology was used to completely sequence the chloroplast genome of *C*. *japonica* and compared with the previously reported chloroplast genomes in *Camellia*. The chloroplast genomes of *C*. *japonica* displayed the typical quadripartite structure of flowering plants, were conservative in gene order and gene content, in comparison with the most lineages of angiosperms. The chloroplast genome sizes ranged from 156,607 to 157,166 bp in length. IR regions are considered as the most conserved region, which considered to be the primary mechanisms affecting length variation of angiosperm chloroplast genomes. Only minor variations were detected at the SC/IR boundaries of six *Camellia*. Occurrence of indels was the main factor effecting the variation of the length in *Camellia* chloroplast genomes. the *Camellia* chloroplast genomes contained more AT content than GC content, which is a common phenomenon in higher plant chloroplast genomes [59–61].

SSRs are widely used in phylogenetic analyses and population genetics and polymorphism investigations. A total of 420 SSR loci were identified and the number of SSRs ranged from 67 to74 in *Camellia*. the mono-nucleotide repeats are the most common SSRs in chloroplast genomes, which make more contributions to the genetic variation than the longer SSRs. Since the structure of chloroplast genomes are conservative, SSR primers are transferable across species and genera. Information involving SSRs in this study will provide useful sources for estimating the phylogenetic relationships among species and genera.

### Potential cpDNA barcodes

*Camellia* is the largest genus in its family, including more than 280 species all over the world. For effective exploration, conservation, and domestication, accurately identified wild species would provide a clear genetic background of this genus. However, the taxonomic inventory of genus *Camellia* is still under controversial, because of the vast amount of species with extensive global distribution and interspecific hybridization. DNA barcoding has been widely used in identify unknown species [66]. The *rbcL* and *matK* is considered as core universal DNA barcodes in many species. Therefore, genomic comparative researches of more complete chloroplast genome sequences have become necessary for developing variable DNA barcodes. These mutation “hotspot” regions can be used to develop novel DNA barcodes [67]. The six potential mutational hotspots (trnS-trnR, petN-psbM, trnF-ndhJ, petA-psbJ, rpl32-trnL, ycf1) identified in this study could be suitable barcodes for plant classification in *Camillia*. In previous reports, the gene ycf1 was recommended as core DNA barcode for plants because of the high divergence [68]. Ycf1 gene has been widely applied in plant phylogeny and DNA barcoding studies [69–70].

Recently, using the chloroplast genome as a super-barcode for plant species identification was discussed [71]. The analyses on chloroplast genome sequence divergence showed that it may indeed be useful as a super-barcode for species identification of *Camellia*. Further research is necessary to investigate whether these hyper-variable regions or complete chloroplast genome sequences could be used as reliable and effective DNA barcodes for species of *Camellia*. The results obtained in this study have significant value for future studies on global genetic diversity assessment, phylogeny, and population genetics of *Camellia*.

### Perspectives of persimmon research in future

It is important to elucidate the genetic relationship of *Camellia* taxa for germplasm conservation, breeding strategies of *Camellia*. The accurate classification of sect. *Thea* have widely been acknowledged to be complex. For example, the taxonomy of *C*. *pubicosta* still has a dispute. Min et al considered that the *C*. *pubicosta* belongs to sect. *Corallina* [71], while Chang and Huang insisted it belongs to sect. *Thea*. [29, 72]. In our research the *C*. *pubicosta* was close to *C*. *sinensis* and *C*. *grandibracteata* supporting *C*. *pubicosta* might be classified into sect. *Thea*. Previous studies reported that species of sect. *Thea* can be divided into two groups, agreeing with the locule ovary number [73,74]. However, our results showed that the classification of this species was not entirely consistent with previous studies [74,75]. For instance, the *C*. *taliensis* and *C*. *cuspidate*, *C*. *grandibracteata* and *C*. *sinensis* were supported as monophyletic respectively. However, the *C*. *taliensis* and *C*. *grandibracteata* have 5 ovaries, while *C*. *cuspidata* and *C*. *sinensis* have 3 ovaries.

The *C*. *japonica* population in Qingdao, Shandong province is the only one in temperate areas in China. While this population has been present in this area since the tertiary, after the quaternary glacier most thermophyilic species extinction or migration to warmer regions. In contrast, *C*. *japonica* adapted to temperate climate. Since then, it has evolved independently and no gene exchanges with the distribution center species. Zhang et al considered that the *C*. *japonica* was the relative evolutionary species. The results of phylogenetic analysis support that *C*. *japonica* and *C*.*oleifera* as monophyletic, However the *C*. *japonica* have 2–3 ovaries and the *C*.*oleifera* have 3–5 ovaries. Our results indicated that the classification of *Camellia* species using locule ovary number may be reconsidered. The combination of traditional classification methods, molecular markers and sequencing of more complete cp genomes of *Camellia* are necessary to solve the problem of *Camellia* classification in the future research.

### Conclusions

We reported the complete chloroplast genome sequences of *C*. *japonica* were reported based on the Illumina HiSeq X Ten platform. *C*. *japonica* chloroplast genomes exhibited a typical quadripartite and circular structure with 156607bp.We investigated the variation of repeat sequences, SSRs among the six complete *Camellia* cp genomes. Selection pressure analysis revealed the influence of different environmental pressures on different *Camellia* chloroplast genomes during long-term evolution. Obvious codon preferences were shown in almost all protein-coding cDNA and amino acid sequences. Lower divergence levels were exhibited in IR and Coding regions than in SC and Non-coding regions, respectively. The results of phylogenetic showed that *C*. *japonica* has the closest relationship with *C*. *oleifera*. Therefore, chloroplast genome resources will be helpful for taxonomic studies, conservation, and breeding programs of the genus *Camellia*.

## Supporting information

S1 Table Features of SSRs in each of the six *Camellia* chloroplast genomes. (DOCX)

S2 Table Distribution of each SSR type in each of the six *Camellia* chloroplast genomes. (DOCX)
