# Supplementary material for: Complete chloroplast genome of Camellia japonica genome structures, comparative and phylogenetic analysis
Source: PLoS One. 2019 May 9;14(5):e0216645. doi: 10.1371/journal.pone.0216645 (PMC6508735; doi:10.1371/journal.pone.0216645)
Supplement: S1 Table — (DOCX) [file pone.0216645.s001.docx]

**TABLE S1 |** **Features of SSRs in each of the six *Camellia* chloroplast genomes.**

| **Taxa** | **SSR type** | **SSR** | **Size** | **Start** | **End** |  | **Locus** | **Location** | **Region** |
| --- | --- | --- | --- | --- | --- | --- | --- | --- | --- |
| *C.japonica* | p1 | **(A)12** | 12 | 349 | 360 |  | *trnH-GUG_psbA* | intergentic | LSC |
| *C.japonica* | p1 | **(A)10** | 10 | 3783 | 3792 |  | *matK_trnK-UUU* | intergentic | LSC |
| *C.japonica* | p1 | **(T)10** | 10 | 6914 | 6923 |  | *rps16_trnQ-UUG* | intergentic | LSC |
| *C.japonica* | p1 | **(T)10** | 10 | 8746 | 8755 |  | *psbI* | gene | LSC |
| *C.japonica* | p1 | **(A)12** | 12 | 8888 | 8899 |  |  |  | LSC |
| *C.japonica* | p1 | **(A)11** | 11 | 9413 | 9423 |  | *trnS-GCU_trnR-UCU* | intergentic | LSC |
| *C.japonica* | p1 | **(A)11** | 11 | 12551 | 12561 |  | *atpA_atpH* | intergentic | LSC |
| *C.japonica* | p1 | **(T)12** | 12 | 13923 | 13934 |  | *atpA_atpH* | intergentic | LSC |
| *C.japonica* | p1 | **(T)13** | 13 | 15055 | 15067 |  | *atpH_atpI* | intergentic | LSC |
| *C.japonica* | p1 | **(A)11** | 11 | 16876 | 16886 |  | *rps2_rpoC2* | intergentic | LSC |
| *C.japonica* | p1 | **(T)11** | 11 | 19088 | 19098 |  | *rpoC2* | gene | LSC |
| *C.japonica* | p1 | **(T)10** | 10 | 26788 | 26797 |  | *rpoB* | gene | LSC |
| *C.japonica* | p1 | **(A)12** | 12 | 32206 | 32217 |  | *trnE-UUC_trnT-GGU* | intergentic | LSC |
| *C.japonica* | p1 | **(A)11** | 11 | 32540 | 32550 |  | *trnE-UUC_trnT-GGU* | intergentic | LSC |
| *C.japonica* | p1 | **(T)13** | 13 | 32844 | 32856 |  | *trnT-GGU_psbD* | intergentic | LSC |
| *C.japonica* | p1 | **(A)14** | 14 | 37238 | 37251 |  | *trnS-UGA_psbZ* | intergentic | LSC |
| *C.japonica* | p1 | **(A)10** | 10 | 37878 | 37887 |  | *psbZ_trnG-UCC* | intergentic | LSC |
| *C.japonica* | p1 | **(A)11** | 11 | 38110 | 38120 |  | *trnG-UCC_trnM-CAU* | intergentic | LSC |
| *C.japonica* | p1 | **(A)12** | 12 | 45854 | 45865 |  | *ycf3_intron* | intron | LSC |
| *C.japonica* | p1 | **(A)12** | 12 | 46110 | 46121 |  | *ycf3_trnS-GGA* | intergentic | LSC |
| *C.japonica* | p1 | **(T)10** | 10 | 48549 | 48558 |  | *trnT-UGU_trnL-UAA* | intergentic | LSC |
| *C.japonica* | p1 | **(A)12** | 12 | 48961 | 48972 |  | *trnT-UGU_trnL-UAA* | intergentic | LSC |
| *C.japonica* | p1 | **(T)11** | 11 | 52277 | 52287 |  | *ndhK* | gene | LSC |
| *C.japonica* | p1 | **(T)10** | 10 | 52864 | 52873 |  | *ndhC_trnV-UAC* | intergentic | LSC |
| *C.japonica* | p1 | **(T)15** | 15 | 53016 | 53030 |  | *ndhC_trnV-UAC* | intergentic | LSC |
| *C.japonica* | p1 | **(T)10** | 10 | 55979 | 55988 |  | *atpB* | gene | LSC |
| *C.japonica* | p1 | **(T)12** | 12 | 56414 | 56425 |  | *atpB_rbcL* | intergentic | LSC |
| *C.japonica* | p1 | **(T)12** | 12 | 58628 | 58639 |  | *rbcL_accD* | intergentic | LSC |
| *C.japonica* | p1 | **(T)12** | 12 | 60408 | 60419 |  | *accD_psaI* | intergentic | LSC |
| *C.japonica* | p1 | **(T)10** | 10 | 62779 | 62788 |  | *ycf4_cemA* | intergentic | LSC |
| *C.japonica* | p1 | **(A)11** | 11 | 64954 | 64964 |  | *petA_psbJ* | intergentic | LSC |
| *C.japonica* | p1 | **(T)12** | 12 | 72910 | 72921 |  | *clpP_intron* | intron | LSC |
| *C.japonica* | p1 | **(T)10** | 10 | 80268 | 80277 |  | *rpoA* | gene | LSC |
| *C.japonica* | p1 | **(T)10** | 10 | 82184 | 82193 |  | *infA_rps8* | intergentic | LSC |
| *C.japonica* | p1 | **(T)13** | 13 | 82709 | 82721 |  | *rps8_rpl14* | intergentic | LSC |
| *C.japonica* | p1 | **(A)11** | 11 | 83235 | 83245 |  | *rpl14_rpl16* | intergentic | LSC |
| *C.japonica* | p1 | **(T)10** | 10 | 84685 | 84694 |  | *rpl16_rps3* | intergentic | LSC |
| *C.japonica* | p1 | **(T)11** | 11 | 104954 | 104964 |  | *trnI-GAU_intron* | intron | IRa |
| *C.japonica* | p1 | **(A)11** | 11 | 110013 | 110023 |  | *rrn5S_trnR-ACG* | intergentic | IRa |
| *C.japonica* | p1 | **(T)15** | 15 | 114932 | 114946 |  | *ndhF_rpl32* | intergentic | SSC |
| *C.japonica* | p1 | **(T)11** | 11 | 116460 | 116470 |  | *trnL-UAG_ccsA* | intergentic | SSC |
| *C.japonica* | p1 | **(A)11** | 11 | 116747 | 116757 |  | *ccsA* | gene | SSC |
| *C.japonica* | p1 | **(T)13** | 13 | 120946 | 120958 |  | *ndhG_ndhI* | intergentic | SSC |
| *C.japonica* | p1 | **(T)12** | 12 | 126932 | 126943 |  | *ycf1* | gene | SSC |
| *C.japonica* | p1 | **(T)12** | 12 | 128547 | 128558 |  | *ycf1* | gene | SSC |
| *C.japonica* | p1 | **(A)12** | 12 | 128851 | 128862 |  | *ycf1* | gene | SSC |
| *C.japonica* | p1 | **(A)10** | 10 | 129384 | 129393 |  | *ycf1* | gene | SSC |
| *C.japonica* | p1 | **(T)17** | 17 | 129765 | 129781 |  | *ycf1* | gene | SSC |
| *C.japonica* | p1 | **(T)11** | 11 | 132843 | 132853 |  | *trnR-ACG_rrn5S* | intergentic | IRb |
| *C.japonica* | p1 | **(A)11** | 11 | 137902 | 137912 |  | *trnI-GAU_intron* | intron | IRb |
| *C.azalea* | p1 | **(A)13** | 13 | 356 | 368 |  | *trnH-GUG_psbA* | intergentic | LSC |
| *C.azalea* | p1 | **(T)12** | 12 | 8762 | 8773 |  | *psbK_psbI* | intergentic | LSC |
| *C.azalea* | p1 | **(A)10** | 10 | 8906 | 8915 |  |  |  | LSC |
| *C.azalea* | p1 | **(A)10** | 10 | 9423 | 9432 |  | *trnS-GCU_trnG-GCC* | intergentic | LSC |
| *C.azalea* | p1 | **(A)16** | 16 | 12550 | 12565 |  | *atpA_atpF* | intergentic | LSC |
| *C.azalea* | p1 | **(T)13** | 13 | 13927 | 13939 |  | *atpF_atpH* | intergentic | LSC |
| *C.azalea* | p1 | **(T)11** | 11 | 15060 | 15070 |  | *atpH_atpI* | intergentic | LSC |
| *C.azalea* | p1 | **(T)12** | 12 | 15459 | 15470 |  | *atpH_atpI* | intergentic | LSC |
| *C.azalea* | p1 | **(A)11** | 11 | 17279 | 17289 |  | *rps2_rpoC2* | intergentic | LSC |
| *C.azalea* | p1 | **(T)11** | 11 | 19485 | 19495 |  | *rpoC2* | gene | LSC |
| *C.azalea* | p1 | **(T)10** | 10 | 27185 | 27194 |  | *rpoB* | gene | LSC |
| *C.azalea* | p1 | **(A)15** | 15 | 32600 | 32614 |  | *trnE-UUC_trnT-GGU* | intergentic | LSC |
| *C.azalea* | p1 | **(A)10** | 10 | 32931 | 32940 |  | *trnE-UUC_trnT-GGU* | intergentic | LSC |
| *C.azalea* | p1 | **(T)13** | 13 | 33234 | 33246 |  | *trnT-GGU_psbD* | intergentic | LSC |
| *C.azalea* | p1 | **(A)13** | 13 | 37629 | 37641 |  | *trnS-UGA_psbZ* | intergentic | LSC |
| *C.azalea* | p1 | **(A)11** | 11 | 38269 | 38279 |  | *psbZ_trnG-UCC* | intergentic | LSC |
| *C.azalea* | p1 | **(A)12** | 12 | 38502 | 38513 |  | *trnG-UCC_trnfM-CAU* | intergentic | LSC |
| *C.azalea* | p1 | **(A)11** | 11 | 44183 | 44193 |  | *psaA_ycf3* | intergentic | LSC |
| *C.azalea* | p1 | **(A)14** | 14 | 46235 | 46248 |  | *ycf3_intron* | intron | LSC |
| *C.azalea* | p1 | **(A)14** | 14 | 46493 | 46506 |  | *ycf3_trnS-GGA* | intergentic | LSC |
| *C.azalea* | p1 | **(A)12** | 12 | 49352 | 49363 |  | *trnT-UGU_trnL-UAA* | intergentic | LSC |
| *C.azalea* | p1 | **(T)11** | 11 | 52651 | 52661 |  | *ndhK* | gene | LSC |
| *C.azalea* | p1 | **(T)10** | 10 | 53238 | 53247 |  | *ndhC_trnV-UAC* | intergentic | LSC |
| *C.azalea* | p1 | **(T)15** | 15 | 53390 | 53404 |  | *ndhC_trnV-UAC* | intergentic | LSC |
| *C.azalea* | p1 | **(T)10** | 10 | 56353 | 56362 |  | *atpB* | gene | LSC |
| *C.azalea* | p1 | **(T)10** | 10 | 56739 | 56748 |  | *atpB_rbcL* | intergentic | LSC |
| *C.azalea* | p1 | **(T)12** | 12 | 56789 | 56800 |  | *atpB_rbcL* | intergentic | LSC |
| *C.azalea* | p1 | **(T)14** | 14 | 59003 | 59016 |  | *rbcL_accD* | intergentic | LSC |
| *C.azalea* | p1 | **(T)14** | 14 | 60784 | 60797 |  | *accD_psaI* | intergentic | LSC |
| *C.azalea* | p1 | **(A)10** | 10 | 65330 | 65339 |  | *petA_psbJ* | intergentic | LSC |
| *C.azalea* | p1 | **(T)10** | 10 | 65468 | 65477 |  | *petA_psbJ* | intergentic | LSC |
| *C.azalea* | p1 | **(T)11** | 11 | 73315 | 73325 |  | *clpP_intron* | intron | LSC |
| *C.azalea* | p1 | **(T)10** | 10 | 80678 | 80687 |  | *rpoA* | gene | LSC |
| *C.azalea* | p1 | **(T)10** | 10 | 82600 | 82609 |  | *infA_rps8* | intergentic | LSC |
| *C.azalea* | p1 | **(T)15** | 15 | 83125 | 83139 |  | *rps8_rpl14* | intergentic | LSC |
| *C.azalea* | p1 | **(A)12** | 12 | 83653 | 83664 |  | *rpl14_rpl16* | intergentic | LSC |
| *C.azalea* | p1 | **(T)12** | 12 | 85099 | 85110 |  | *rpl16_intron* | intron | LSC |
| *C.azalea* | p1 | **(T)10** | 10 | 105352 | 105361 |  | *trnI-GAU_intron* | intron | IRa |
| *C.azalea* | p1 | **(A)11** | 11 | 110410 | 110420 |  | *rrn5_trnR-ACG* | intergentic | IRa |
| *C.azalea* | p1 | **(T)13** | 13 | 115416 | 115428 |  | *ndhF_rpl32* | intergentic | SSC |
| *C.azalea* | p1 | **(T)10** | 10 | 116943 | 116952 |  | *trnL-UAG_ccsA* | intergentic | SSC |
| *C.azalea* | p1 | **(A)11** | 11 | 117229 | 117239 |  | *ccsA* | gene | SSC |
| *C.azalea* | p1 | **(T)14** | 14 | 121428 | 121441 |  | *ndhG_ndhI* | intergentic | SSC |
| *C.azalea* | p1 | **(T)12** | 12 | 127395 | 127406 |  | *ycf1* | gene | SSC |
| *C.azalea* | p1 | **(T)12** | 12 | 129010 | 129021 |  | *ycf1* | gene | SSC |
| *C.azalea* | p1 | **(A)12** | 12 | 129314 | 129325 |  | *ycf1* | gene | SSC |
| *C.azalea* | p1 | **(A)10** | 10 | 129847 | 129856 |  | *ycf1* | gene | SSC |
| *C.azalea* | p1 | **(T)17** | 17 | 130228 | 130244 |  | *ycf1* | gene | SSC |
| *C.azalea* | p1 | **(T)11** | 11 | 133294 | 133304 |  | *trnR-ACG_rrn5* | intergentic | IRb |
| *C.azalea* | p1 | **(A)10** | 10 | 138353 | 138362 |  | *trnI-GAU_intron* | intron | IRb |
| *C.crapnelliana* | p1 | **(A)12** | 12 | 356 | 367 |  | *trnH-GUG_psbA* | intergentic | LSC |
| *C.crapnelliana* | p1 | **(A)10** | 10 | 3790 | 3799 |  | *matK_trnK-UUU* | intergentic | LSC |
| *C.crapnelliana* | p1 | **(T)12** | 12 | 8744 | 8755 |  | *psbI* | gene | LSC |
| *C.crapnelliana* | p1 | **(A)12** | 12 | 8888 | 8899 |  |  |  | LSC |
| *C.crapnelliana* | p1 | **(A)14** | 14 | 9413 | 9426 |  | *trnS-GCU_trnR-UCU* | intergentic | LSC |
| *C.crapnelliana* | p1 | **(A)11** | 11 | 12545 | 12555 |  | *atpA_atpF* | intergentic | LSC |
| *C.crapnelliana* | p1 | **(T)12** | 12 | 13917 | 13928 |  | *atpF_atpH* | intergentic | LSC |
| *C.crapnelliana* | p1 | **(T)12** | 12 | 15049 | 15060 |  | *atpH_atpI* | intergentic | LSC |
| *C.crapnelliana* | p1 | **(T)13** | 13 | 15449 | 15461 |  | *atpH_atpI* | intergentic | LSC |
| *C.crapnelliana* | p1 | **(A)12** | 12 | 17270 | 17281 |  | *rps2_rpoC2* | intergentic | LSC |
| *C.crapnelliana* | p1 | **(T)11** | 11 | 19477 | 19487 |  | *rpoC2* | gene | LSC |
| *C.crapnelliana* | p1 | **(T)10** | 10 | 27171 | 27180 |  | *rpoB* | gene | LSC |
| *C.crapnelliana* | p1 | **(A)17** | 17 | 32580 | 32596 |  | *trnE-UUC_trnT-GGU* | intergentic | LSC |
| *C.crapnelliana* | p1 | **(A)11** | 11 | 32913 | 32923 |  | *trnE-UUC_trnT-GGU* | intergentic | LSC |
| *C.crapnelliana* | p1 | **(T)13** | 13 | 33217 | 33229 |  | *trnM-CAU_psbD* | intergentic | LSC |
| *C.crapnelliana* | p1 | **(T)10** | 10 | 33572 | 33581 |  | *trnM-CAU_psbD* | intergentic | LSC |
| *C.crapnelliana* | p1 | **(T)10** | 10 | 37213 | 37222 |  | *psbC_trnS-UGA* | intergentic | LSC |
| *C.crapnelliana* | p1 | **(A)13** | 13 | 37613 | 37625 |  | *trnS-UGA_psbZ* | intergentic | LSC |
| *C.crapnelliana* | p1 | **(A)11** | 11 | 38252 | 38262 |  | *psbZ_trnG-UCC* | intergentic | LSC |
| *C.crapnelliana* | p1 | **(A)11** | 11 | 38485 | 38495 |  | *trnG-UCC_trnfM-CAU* | intergentic | LSC |
| *C.crapnelliana* | p1 | **(A)11** | 11 | 44170 | 44180 |  | *psaA_ycf3* | intergentic | LSC |
| *C.crapnelliana* | p1 | **(A)12** | 12 | 46221 | 46232 |  | *ycf3_intron* | intron | LSC |
| *C.crapnelliana* | p1 | **(A)11** | 11 | 46477 | 46487 |  | *ycf3_trnS-GGA* | intergentic | LSC |
| *C.crapnelliana* | p1 | **(T)10** | 10 | 48921 | 48930 |  | *trnT-UGU_trnL-UAA* | intergentic | LSC |
| *C.crapnelliana* | p1 | **(A)12** | 12 | 49333 | 49344 |  | *trnT-UGU_trnL-UAA* | intergentic | LSC |
| *C.crapnelliana* | p1 | **(T)10** | 10 | 52632 | 52641 |  | *ndhK_ndhC* | intergentic | LSC |
| *C.crapnelliana* | p1 | **(T)10** | 10 | 53218 | 53227 |  | *ndhC_trnV-UAC* | intergentic | LSC |
| *C.crapnelliana* | p1 | **(T)13** | 13 | 53370 | 53382 |  | *ndhC_trnV-UAC* | intergentic | LSC |
| *C.crapnelliana* | p1 | **(T)10** | 10 | 56331 | 56340 |  | *atpB* | gene | LSC |
| *C.crapnelliana* | p1 | **(T)13** | 13 | 56766 | 56778 |  | *atpB_rbcL* | intergentic | LSC |
| *C.crapnelliana* | p1 | **(T)15** | 15 | 58981 | 58995 |  | *rbcL_accD* | intergentic | LSC |
| *C.crapnelliana* | p1 | **(T)14** | 14 | 60763 | 60776 |  | *accD_psaI* | intergentic | LSC |
| *C.crapnelliana* | p1 | **(T)10** | 10 | 63136 | 63145 |  | *ycf4_cemA* | intergentic | LSC |
| *C.crapnelliana* | p1 | **(A)11** | 11 | 65311 | 65321 |  | *petA_psbJ* | intergentic | LSC |
| *C.crapnelliana* | p1 | **(T)14** | 14 | 65763 | 65776 |  | *petA_psbJ* | intergentic | LSC |
| *C.crapnelliana* | p1 | **(T)14** | 14 | 73284 | 73297 |  | *clpP_intron* | intron | LSC |
| *C.crapnelliana* | p1 | **(T)10** | 10 | 80651 | 80660 |  | *rpoA* | gene | LSC |
| *C.crapnelliana* | p1 | **(T)10** | 10 | 82573 | 82582 |  | *infA_rps8* | intergentic | LSC |
| *C.crapnelliana* | p1 | **(T)17** | 17 | 83098 | 83114 |  | *rps8_rpl14* | intergentic | LSC |
| *C.crapnelliana* | p1 | **(A)14** | 14 | 83628 | 83641 |  | *rpl14_rpl16* | intergentic | LSC |
| *C.crapnelliana* | p1 | **(T)11** | 11 | 85081 | 85091 |  | *rpl16_intron* | intron | LSC |
| *C.crapnelliana* | p1 | **(T)10** | 10 | 105352 | 105361 |  | *trnI-GAU_intron* | intron | IRa |
| *C.crapnelliana* | p1 | **(A)12** | 12 | 110410 | 110421 |  | *rrn5_trnR-ACG* | intergentic | IRa |
| *C.crapnelliana* | p1 | **(T)14** | 14 | 115335 | 115348 |  | *ndhF_rpl32* | intergentic | SSC |
| *C.crapnelliana* | p1 | **(T)10** | 10 | 116862 | 116871 |  | *trnL-UAG_ccsA* | intergentic | SSC |
| *C.crapnelliana* | p1 | **(A)11** | 11 | 117148 | 117158 |  | *ccsA* | gene | SSC |
| *C.crapnelliana* | p1 | **(T)10** | 10 | 117976 | 117985 |  | *ccsA_ndhD* | intergentic | SSC |
| *C.crapnelliana* | p1 | **(T)12** | 12 | 121348 | 121359 |  | *ndhG_ndhI* | intergentic | SSC |
| *C.crapnelliana* | p1 | **(T)12** | 12 | 127321 | 127332 |  | *ycf1* | gene | SSC |
| *C.crapnelliana* | p1 | **(T)12** | 12 | 128936 | 128947 |  | *ycf1* | gene | SSC |
| *C.crapnelliana* | p1 | **(A)12** | 12 | 129240 | 129251 |  | *ycf1* | gene | SSC |
| *C.crapnelliana* | p1 | **(A)10** | 10 | 129773 | 129782 |  | *ycf1* | gene | SSC |
| *C.crapnelliana* | p1 | **(T)17** | 17 | 130154 | 130170 |  | *ycf1* | gene | SSC |
| *C.crapnelliana* | p1 | **(T)12** | 12 | 133232 | 133243 |  | *trnR-ACG_rrn5* | intergentic | IRb |
| *C.crapnelliana* | p1 | **(A)10** | 10 | 138292 | 138301 |  | *trnI-GAU_intron* | intron | IRb |
| *C.huana* | p1 | **(A)13** | 13 | 356 | 368 |  | *trnH (GUG)_psbA* | intergentic | LSC |
| *C.huana* | p1 | **(A)10** | 10 | 3786 | 3795 |  | *matK_trnK (UUU)* | intergentic | LSC |
| *C.huana* | p1 | **(A)10** | 10 | 5493 | 5502 |  | *rps16_intron* | intron | LSC |
| *C.huana* | p1 | **(T)11** | 11 | 8751 | 8761 |  | *psbK_psbI* | intergentic | LSC |
| *C.huana* | p1 | **(A)10** | 10 | 8894 | 8903 |  |  |  | LSC |
| *C.huana* | p1 | **(A)16** | 16 | 12542 | 12557 |  | *atpA_atpF* | intergentic | LSC |
| *C.huana* | p1 | **(T)12** | 12 | 13919 | 13930 |  | *atpF_atpH* | intergentic | LSC |
| *C.huana* | p1 | **(T)11** | 11 | 15046 | 15056 |  | *atpH_atpI* | intergentic | LSC |
| *C.huana* | p1 | **(T)12** | 12 | 15445 | 15456 |  | *atpH_atpI* | intergentic | LSC |
| *C.huana* | p1 | **(A)10** | 10 | 17265 | 17274 |  | *rps2_rpoC2* | intergentic | LSC |
| *C.huana* | p1 | **(T)11** | 11 | 19470 | 19480 |  | *rpoC2* | gene | LSC |
| *C.huana* | p1 | **(T)10** | 10 | 27170 | 27179 |  | *rpoB* | gene | LSC |
| *C.huana* | p1 | **(T)10** | 10 | 32467 | 32476 |  | *trnE (UUC)_trnT (GGU)* | intergentic | LSC |
| *C.huana* | p1 | **(A)14** | 14 | 32590 | 32603 |  | *trnE (UUC)_trnT (GGU)* | intergentic | LSC |
| *C.huana* | p1 | **(A)10** | 10 | 32920 | 32929 |  | *trnE (UUC)_trnT (GGU)* | intergentic | LSC |
| *C.huana* | p1 | **(T)11** | 11 | 33223 | 33233 |  | *trnT (GGU)_psbD* | intergentic | LSC |
| *C.huana* | p1 | **(T)10** | 10 | 34007 | 34016 |  | *trnT (GGU)_psbD* | intergentic | LSC |
| *C.huana* | p1 | **(T)10** | 10 | 37216 | 37225 |  | *psbC_trnS (UGA)* | intergentic | LSC |
| *C.huana* | p1 | **(A)13** | 13 | 37616 | 37628 |  | *trnS (UGA)_psbZ* | intergentic | LSC |
| *C.huana* | p1 | **(A)11** | 11 | 38256 | 38266 |  | *psbZ_trnG (GCC)* | intergentic | LSC |
| *C.huana* | p1 | **(A)11** | 11 | 38489 | 38499 |  | *trnG (GCC)_tRNA-fM (CAU)* | intergentic | LSC |
| *C.huana* | p1 | **(A)10** | 10 | 44169 | 44178 |  | *psaA_ycf3* | intergentic | LSC |
| *C.huana* | p1 | **(A)11** | 11 | 46209 | 46219 |  | *ycf3_intron* | intron | LSC |
| *C.huana* | p1 | **(A)14** | 14 | 46464 | 46477 |  | *ycf3_trnS (GGA)* | intergentic | LSC |
| *C.huana* | p1 | **(A)12** | 12 | 49290 | 49301 |  | *trnT (UGU)_trnL (UAA)* | intergentic | LSC |
| *C.huana* | p1 | **(T)11** | 11 | 52584 | 52594 |  | *ndhK* | gene | LSC |
| *C.huana* | p1 | **(T)13** | 13 | 53321 | 53333 |  | *ndhC_trnV (UAC)* | intergentic | LSC |
| *C.huana* | p1 | **(T)10** | 10 | 56282 | 56291 |  | *atpB* | gene | LSC |
| *C.huana* | p1 | **(T)13** | 13 | 56715 | 56727 |  | *atpB_rbcL* | intergentic | LSC |
| *C.huana* | p1 | **(T)15** | 15 | 58925 | 58939 |  | *rbcL_accD* | intergentic | LSC |
| *C.huana* | p1 | **(T)14** | 14 | 60707 | 60720 |  | *accD_psaI* | intergentic | LSC |
| *C.huana* | p1 | **(T)10** | 10 | 63079 | 63088 |  | *ycf4_cemA* | intergentic | LSC |
| *C.huana* | p1 | **(A)10** | 10 | 65240 | 65249 |  | *petA_psbJ* | intergentic | LSC |
| *C.huana* | p1 | **(T)10** | 10 | 65378 | 65387 |  | *petA_psbJ* | intergentic | LSC |
| *C.huana* | p1 | **(T)10** | 10 | 65694 | 65703 |  | *petA_psbJ* | intergentic | LSC |
| *C.huana* | p1 | **(T)10** | 10 | 70888 | 70897 |  | *rps18_rpl20* | intergentic | LSC |
| *C.huana* | p1 | **(T)11** | 11 | 73201 | 73211 |  | *clpP_intron* | intron | LSC |
| *C.huana* | p1 | **(T)10** | 10 | 80570 | 80579 |  | *rpoA* | gene | LSC |
| *C.huana* | p1 | **(T)10** | 10 | 82492 | 82501 |  | *infA_rps8* | intergentic | LSC |
| *C.huana* | p1 | **(T)14** | 14 | 83017 | 83030 |  | *rps8_rpl14* | intergentic | LSC |
| *C.huana* | p1 | **(A)11** | 11 | 83544 | 83554 |  | *rpl14_rpl16* | intergentic | LSC |
| *C.huana* | p1 | **(T)11** | 11 | 84994 | 85004 |  | *rpl16_intron* | intron | LSC |
| *C.huana* | p1 | **(T)10** | 10 | 86591 | 86600 |  | *rps19* | gene | IRa |
| *C.huana* | p1 | **(T)10** | 10 | 105282 | 105291 |  | *trnI (GAU)_intron* | intron | IRa |
| *C.huana* | p1 | **(A)11** | 11 | 110340 | 110350 |  | *rrn5_trnR (ACG)* | intergentic | IRa |
| *C.huana* | p1 | **(T)13** | 13 | 115256 | 115268 |  | *ndhF_rpl32* | intergentic | SSC |
| *C.huana* | p1 | **(A)11** | 11 | 117068 | 117078 |  | *ccsA* | gene | SSC |
| *C.huana* | p1 | **(T)11** | 11 | 121267 | 121277 |  | *ndhG_ndhI* | intergentic | SSC |
| *C.huana* | p1 | **(T)12** | 12 | 127232 | 127243 |  | *ycf1* | gene | SSC |
| *C.huana* | p1 | **(T)12** | 12 | 128847 | 128858 |  | *ycf1* | gene | SSC |
| *C.huana* | p1 | **(A)12** | 12 | 129151 | 129162 |  | *ycf1* | gene | SSC |
| *C.huana* | p1 | **(A)10** | 10 | 129684 | 129693 |  | *ycf1* | gene | SSC |
| *C.huana* | p1 | **(T)17** | 17 | 130065 | 130081 |  | *ycf1* | gene | SSC |
| *C.huana* | p1 | **(T)11** | 11 | 133122 | 133132 |  | *trnR (ACG)_rrn5* | intergentic | IRb |
| *C.huana* | p1 | **(A)10** | 10 | 138181 | 138190 |  | *trnI (GAU)_intron* | intron | IRb |
| *C.huana* | p1 | **(A)10** | 10 | 156872 | 156881 |  | *rpl2_trnH (GUG)* | intergentic | IRb |
| *C.liberofilamenta* | p1 | **(A)12** | 12 | 356 | 367 |  | *trnH (GUG)_psbA* | intergentic | LSC |
| *C.liberofilamenta* | p1 | **(A)11** | 11 | 5497 | 5507 |  | *rps16_intron* | intron | LSC |
| *C.liberofilamenta* | p1 | **(T)13** | 13 | 8763 | 8775 |  | *psbK_psbI* | intergentic | LSC |
| *C.liberofilamenta* | p1 | **(A)11** | 11 | 8908 | 8918 |  |  |  | LSC |
| *C.liberofilamenta* | p1 | **(A)15** | 15 | 12557 | 12571 |  | *atpA_atpF* | intergentic | LSC |
| *C.liberofilamenta* | p1 | **(T)13** | 13 | 13933 | 13945 |  | *atpF_atpH* | intergentic | LSC |
| *C.liberofilamenta* | p1 | **(T)12** | 12 | 15061 | 15072 |  | *atpH_atpI* | intergentic | LSC |
| *C.liberofilamenta* | p1 | **(T)12** | 12 | 15461 | 15472 |  | *atpH_atpI* | intergentic | LSC |
| *C.liberofilamenta* | p1 | **(A)10** | 10 | 17281 | 17290 |  | *rps2_rpoC2* | intergentic | LSC |
| *C.liberofilamenta* | p1 | **(T)11** | 11 | 19486 | 19496 |  | *rpoC2* | gene | LSC |
| *C.liberofilamenta* | p1 | **(T)10** | 10 | 27186 | 27195 |  | *rpoB* | gene | LSC |
| *C.liberofilamenta* | p1 | **(A)15** | 15 | 32605 | 32619 |  | *trnE (UUC)_trnT (GGU)* | intergentic | LSC |
| *C.liberofilamenta* | p1 | **(A)11** | 11 | 32936 | 32946 |  | *trnE (UUC)_trnT (GGU)* | intergentic | LSC |
| *C.liberofilamenta* | p1 | **(T)12** | 12 | 33240 | 33251 |  | *trnT (GGU)_psbD* | intergentic | LSC |
| *C.liberofilamenta* | p1 | **(T)10** | 10 | 33595 | 33604 |  | *trnT (GGU)_psbD* | intergentic | LSC |
| *C.liberofilamenta* | p1 | **(T)10** | 10 | 37234 | 37243 |  | *psbC_trnS (UGA)* | intergentic | LSC |
| *C.liberofilamenta* | p1 | **(A)14** | 14 | 37634 | 37647 |  | *trnS (UGA)_psbZ* | intergentic | LSC |
| *C.liberofilamenta* | p1 | **(A)11** | 11 | 38275 | 38285 |  | *psbZ_trnG (GCC)* | intergentic | LSC |
| *C.liberofilamenta* | p1 | **(A)12** | 12 | 38459 | 38470 |  | *trnG (GCC)_tRNA-fM (CAU)* | intergentic | LSC |
| *C.liberofilamenta* | p1 | **(A)10** | 10 | 44145 | 44154 |  | *psaA_ycf3* | intergentic | LSC |
| *C.liberofilamenta* | p1 | **(A)12** | 12 | 46185 | 46196 |  | *ycf3_intron* | intron | LSC |
| *C.liberofilamenta* | p1 | **(A)14** | 14 | 46441 | 46454 |  | *ycf3_trnS (GGA)* | intergentic | LSC |
| *C.liberofilamenta* | p1 | **(A)12** | 12 | 49300 | 49311 |  | *trnT (UGU)_trnL (UAA)* | intergentic | LSC |
| *C.liberofilamenta* | p1 | **(T)11** | 11 | 52594 | 52604 |  | *ndhK* | gene | LSC |
| *C.liberofilamenta* | p1 | **(T)14** | 14 | 53331 | 53344 |  | *ndhC_trnV (UAC)* | intergentic | LSC |
| *C.liberofilamenta* | p1 | **(T)10** | 10 | 53449 | 53458 |  | *trnV (UAC)_intron* | intron | LSC |
| *C.liberofilamenta* | p1 | **(T)10** | 10 | 56294 | 56303 |  | *atpB* | gene | LSC |
| *C.liberofilamenta* | p1 | **(T)13** | 13 | 56727 | 56739 |  | *atpB_rbcL* | intergentic | LSC |
| *C.liberofilamenta* | p1 | **(T)14** | 14 | 58942 | 58955 |  | *rbcL_accD* | intergentic | LSC |
| *C.liberofilamenta* | p1 | **(T)15** | 15 | 60723 | 60737 |  | *accD_psaI* | intergentic | LSC |
| *C.liberofilamenta* | p1 | **(A)11** | 11 | 65269 | 65279 |  | *petA_psbJ* | intergentic | LSC |
| *C.liberofilamenta* | p1 | **(T)10** | 10 | 65408 | 65417 |  | *petA_psbJ* | intergentic | LSC |
| *C.liberofilamenta* | p1 | **(T)11** | 11 | 65732 | 65742 |  | *petA_psbJ* | intergentic | LSC |
| *C.liberofilamenta* | p1 | **(T)10** | 10 | 70910 | 70919 |  | *rps18_rpl20* | intergentic | LSC |
| *C.liberofilamenta* | p1 | **(T)10** | 10 | 73219 | 73228 |  | *clpP_intron* | intron | LSC |
| *C.liberofilamenta* | p1 | **(T)10** | 10 | 80581 | 80590 |  | *rpoA* | gene | LSC |
| *C.liberofilamenta* | p1 | **(T)10** | 10 | 82503 | 82512 |  | *infA_rps8* | intergentic | LSC |
| *C.liberofilamenta* | p1 | **(T)15** | 15 | 83028 | 83042 |  | *rps8_rpl14* | intergentic | LSC |
| *C.liberofilamenta* | p1 | **(A)10** | 10 | 83556 | 83565 |  | *rpl14_rpl16* | intergentic | LSC |
| *C.liberofilamenta* | p1 | **(T)11** | 11 | 85005 | 85015 |  | *rpl16_intron* | intron | LSC |
| *C.liberofilamenta* | p1 | **(T)10** | 10 | 105274 | 105283 |  | *trnI (GAU)_intron* | intron | IRa |
| *C.liberofilamenta* | p1 | **(A)11** | 11 | 110332 | 110342 |  | *rrn5_trnR (ACG)* | intergentic | IRa |
| *C.liberofilamenta* | p1 | **(T)14** | 14 | 115253 | 115266 |  | *ndhF_rpl32* | intergentic | SSC |
| *C.liberofilamenta* | p1 | **(A)11** | 11 | 117048 | 117058 |  | *ccsA* | gene | SSC |
| *C.liberofilamenta* | p1 | **(T)12** | 12 | 121247 | 121258 |  | *ndhG_ndhI* | intergentic | SSC |
| *C.liberofilamenta* | p1 | **(T)12** | 12 | 127212 | 127223 |  | *ycf1* | gene | SSC |
| *C.liberofilamenta* | p1 | **(T)12** | 12 | 128827 | 128838 |  | *ycf1* | gene | SSC |
| *C.liberofilamenta* | p1 | **(A)12** | 12 | 129131 | 129142 |  | *ycf1* | gene | SSC |
| *C.liberofilamenta* | p1 | **(A)10** | 10 | 129664 | 129673 |  | *ycf1* | gene | SSC |
| *C.liberofilamenta* | p1 | **(T)17** | 17 | 130045 | 130061 |  | *ycf1* | gene | SSC |
| *C.liberofilamenta* | p1 | **(T)11** | 11 | 133103 | 133113 |  | *trnR (ACG)_rrn5* | intergentic | IRb |
| *C.liberofilamenta* | p1 | **(A)10** | 10 | 138162 | 138171 |  | *trnI (GAU)_intron* | intron | IRb |
| *C.luteoflora* | p1 | **(A)14** | 14 | 375 | 388 |  | *trnH (GUG)_psbA* | intergentic | LSC |
| *C.luteoflora* | p1 | **(A)10** | 10 | 3811 | 3820 |  | *matK_trnK (UUU)* | intergentic | LSC |
| *C.luteoflora* | p1 | **(A)10** | 10 | 5518 | 5527 |  | *rps16_intron* | intron | LSC |
| *C.luteoflora* | p1 | **(T)11** | 11 | 8782 | 8792 |  | *psbK_psbI* | intergentic | LSC |
| *C.luteoflora* | p1 | **(A)12** | 12 | 8925 | 8936 |  |  |  | LSC |
| *C.luteoflora* | p1 | **(A)16** | 16 | 12573 | 12588 |  | *atpA_atpF* | intergentic | LSC |
| *C.luteoflora* | p1 | **(T)12** | 12 | 13950 | 13961 |  | *atpF_atpH* | intergentic | LSC |
| *C.luteoflora* | p1 | **(T)12** | 12 | 15082 | 15093 |  | *atpH_atpI* | intergentic | LSC |
| *C.luteoflora* | p1 | **(T)12** | 12 | 15482 | 15493 |  | *atpH_atpI* | intergentic | LSC |
| *C.luteoflora* | p1 | **(A)11** | 11 | 17302 | 17312 |  | *rps2_rpoC2* | intergentic | LSC |
| *C.luteoflora* | p1 | **(T)11** | 11 | 19508 | 19518 |  | *rpoC2* | gene | LSC |
| *C.luteoflora* | p1 | **(T)10** | 10 | 27208 | 27217 |  | *rpoB* | gene | LSC |
| *C.luteoflora* | p1 | **(A)15** | 15 | 32627 | 32641 |  | *trnE (UUC)_trnT (GGU)* | intergentic | LSC |
| *C.luteoflora* | p1 | **(A)10** | 10 | 32964 | 32973 |  | *trnE (UUC)_trnT (GGU)* | intergentic | LSC |
| *C.luteoflora* | p1 | **(T)14** | 14 | 33267 | 33280 |  | *trnT (GGU)_psbD* | intergentic | LSC |
| *C.luteoflora* | p1 | **(A)11** | 11 | 37663 | 37673 |  | *trnS (UGA)_psbZ* | intergentic | LSC |
| *C.luteoflora* | p1 | **(A)10** | 10 | 38301 | 38310 |  | *psbZ_trnG (GCC)* | intergentic | LSC |
| *C.luteoflora* | p1 | **(A)14** | 14 | 38532 | 38545 |  | *trnG (GCC)_tRNA-fM (CAU)* | intergentic | LSC |
| *C.luteoflora* | p1 | **(A)11** | 11 | 44213 | 44223 |  | *psaA_ycf3* | intergentic | LSC |
| *C.luteoflora* | p1 | **(A)12** | 12 | 46264 | 46275 |  | *ycf3_intron* | intron | LSC |
| *C.luteoflora* | p1 | **(A)14** | 14 | 46520 | 46533 |  | *ycf3_trnS (GGA)* | intergentic | LSC |
| *C.luteoflora* | p1 | **(A)13** | 13 | 49379 | 49391 |  | *trnT (UGU)_trnL (UAA)* | intergentic | LSC |
| *C.luteoflora* | p1 | **(T)11** | 11 | 52679 | 52689 |  | *ndhK* | gene | LSC |
| *C.luteoflora* | p1 | **(T)10** | 10 | 53266 | 53275 |  | *ndhC_trnV (UAC)* | intergentic | LSC |
| *C.luteoflora* | p1 | **(T)13** | 13 | 53418 | 53430 |  | *ndhC_trnV (UAC)* | intergentic | LSC |
| *C.luteoflora* | p1 | **(T)10** | 10 | 56379 | 56388 |  | *atpB* | gene | LSC |
| *C.luteoflora* | p1 | **(T)13** | 13 | 56813 | 56825 |  | *atpB_rbcL* | intergentic | LSC |
| *C.luteoflora* | p1 | **(T)14** | 14 | 59028 | 59041 |  | *rbcL_accD* | intergentic | LSC |
| *C.luteoflora* | p1 | **(T)14** | 14 | 60809 | 60822 |  | *accD_psaI* | intergentic | LSC |
| *C.luteoflora* | p1 | **(A)10** | 10 | 65356 | 65365 |  | *petA_psbJ* | intergentic | LSC |
| *C.luteoflora* | p1 | **(T)10** | 10 | 65494 | 65503 |  | *petA_psbJ* | intergentic | LSC |
| *C.luteoflora* | p1 | **(T)12** | 12 | 65844 | 65855 |  | *petA_psbJ* | intergentic | LSC |
| *C.luteoflora* | p1 | **(T)10** | 10 | 71045 | 71054 |  | *rps18_rpl20* | intergentic | LSC |
| *C.luteoflora* | p1 | **(T)10** | 10 | 73358 | 73367 |  | *clpP_intron* | intron | LSC |
| *C.luteoflora* | p1 | **(T)10** | 10 | 80720 | 80729 |  | *rpoA* | gene | LSC |
| *C.luteoflora* | p1 | **(T)10** | 10 | 82642 | 82651 |  | *infA_rps8* | intergentic | LSC |
| *C.luteoflora* | p1 | **(T)14** | 14 | 83167 | 83180 |  | *rps8_rpl14* | intergentic | LSC |
| *C.luteoflora* | p1 | **(A)12** | 12 | 83694 | 83705 |  | *rpl14_rpl16* | intergentic | LSC |
| *C.luteoflora* | p1 | **(T)11** | 11 | 85145 | 85155 |  | *rpl16_intron* | intron | LSC |
| *C.luteoflora* | p1 | **(T)10** | 10 | 105415 | 105424 |  | *trnI (GAU)_intron* | intron | IRa |
| *C.luteoflora* | p1 | **(A)11** | 11 | 110473 | 110483 |  | *rrn5_trnR (ACG)* | intergentic | IRa |
| *C.luteoflora* | p1 | **(T)13** | 13 | 115496 | 115508 |  | *ndhF_rpl32* | intergentic | SSC |
| *C.luteoflora* | p1 | **(T)10** | 10 | 117023 | 117032 |  | *trnL (UAG)_ccsA* | intergentic | SSC |
| *C.luteoflora* | p1 | **(A)11** | 11 | 117309 | 117319 |  | *ccsA* | gene | SSC |
| *C.luteoflora* | p1 | **(T)14** | 14 | 121508 | 121521 |  | *ndhG_ndhI* | intergentic | SSC |
| *C.luteoflora* | p1 | **(T)12** | 12 | 127487 | 127498 |  | *ycf1* | gene | SSC |
| *C.luteoflora* | p1 | **(T)12** | 12 | 129102 | 129113 |  | *ycf1* | gene | SSC |
| *C.luteoflora* | p1 | **(A)12** | 12 | 129406 | 129417 |  | *ycf1* | gene | SSC |
| *C.luteoflora* | p1 | **(A)10** | 10 | 129939 | 129948 |  | *ycf1* | gene | SSC |
| *C.luteoflora* | p1 | **(T)17** | 17 | 130320 | 130336 |  | *ycf1* | gene | SSC |
| *C.luteoflora* | p1 | **(T)11** | 11 | 133403 | 133413 |  | *trnR (ACG)_rrn5* | intergentic | IRb |
| *C.luteoflora* | p1 | **(A)10** | 10 | 138462 | 138471 |  | *trnI (GAU)_intron* | intron | IRb |
| *C.japonica* | p2 | **(AT)5** | 10 | 20461 | 20470 |  | *rpoC2* | gene | LSC |
| *C.japonica* | p2 | **(AT)5** | 10 | 83948 | 83957 |  | *rpl16_rps3* | intergentic | LSC |
| *C.japonica* | p2 | **(TA)5** | 10 | 86915 | 86924 |  | *rpl2_intron* | intron | IRa |
| *C.japonica* | p2 | **(AT)5** | 10 | 155941 | 155950 |  | *rpl2_intron* | intron | IRb |
| *C.azalea* | p2 | **(AT)5** | 10 | 20858 | 20867 |  | *rpoC2* | gene | LSC |
| *C.azalea* | p2 | **(AT)5** | 10 | 84367 | 84376 |  | *rpl16_intron* | intron | LSC |
| *C.azalea* | p2 | **(TA)5** | 10 | 87331 | 87340 |  | *rpl2_intron* | intron | IRa |
| *C.azalea* | p2 | **(AT)5** | 10 | 156373 | 156382 |  | *rpl2_intron* | intron | IRb |
| *C.crapnelliana* | p2 | **(AT)5** | 10 | 20850 | 20859 |  | *rpoC2* | gene | LSC |
| *C.crapnelliana* | p2 | **(AT)5** | 10 | 84350 | 84359 |  | *rpl16_intron* | intron | LSC |
| *C.crapnelliana* | p2 | **(TA)5** | 10 | 87318 | 87327 |  | *rpl2_intron* | intron | IRa |
| *C.crapnelliana* | p2 | **(AT)5** | 10 | 156337 | 156346 |  | *rpl2_intron* | intron | IRb |
| *C.huana* | p2 | **(AT)5** | 10 | 20869 | 20878 |  | *rpoC2* | gene | LSC |
| *C.huana* | p2 | **(AT)5** | 10 | 84283 | 84292 |  | *rpl16_intron* | intron | LSC |
| *C.huana* | p2 | **(TA)5** | 10 | 87251 | 87260 |  | *rpl2_intron* | intron | IRa |
| *C.huana* | p2 | **(AT)5** | 10 | 156263 | 156272 |  | *rpl2_intron* | intron | IRb |
| *C.liberofilamenta* | p2 | **(AT)5** | 10 | 20885 | 20894 |  | *rpoC2* | gene | LSC |
| *C.liberofilamenta* | p2 | **(AT)5** | 10 | 84294 | 84303 |  | *rpl16_intron* | intron | LSC |
| *C.liberofilamenta* | p2 | **(TA)5** | 10 | 87262 | 87271 |  | *rpl2_intron* | intron | IRa |
| *C.liberofilamenta* | p2 | **(AT)5** | 10 | 156225 | 156234 |  | *rpl2_intron* | intron | IRb |
| *C.luteoflora* | p2 | **(AT)5** | 10 | 20907 | 20916 |  | *rpoC2* | gene | LSC |
| *C.luteoflora* | p2 | **(AT)5** | 10 | 84434 | 84443 |  | *rpl16_intron* | intron | LSC |
| *C.luteoflora* | p2 | **(TA)5** | 10 | 87402 | 87411 |  | *rpl2_intron* | intron | IRa |
| *C.luteoflora* | p2 | **(AT)5** | 10 | 156526 | 156535 |  | *rpl2_intron* | intron | IRb |
| *C.japonica* | p3 | **(TTC)4** | 12 | 69730 | 69741 |  | *psaJ_rpl33* | intergentic | LSC |
| *C.azalea* | p3 | **(TTC)4** | 12 | 70124 | 70135 |  | *psaJ_rpl33* | intergentic | LSC |
| *C.crapnelliana* | p3 | **(TTC)4** | 12 | 70100 | 70111 |  | *psaJ_rpl33* | intergentic | LSC |
| *C.huana* | p3 | **(TTC)4** | 12 | 70041 | 70052 |  | *psaJ_rpl33* | intergentic | LSC |
| *C.huana* | p3 | **(ATT)4** | 12 | 74728 | 74739 |  | *clpP_psbB* | intergentic | LSC |
| *C.liberofilamenta* | p3 | **(TTC)4** | 12 | 70069 | 70080 |  | *psaJ_rpl33* | intergentic | LSC |
| *C.luteoflora* | p3 | **(TTC)4** | 12 | 70193 | 70204 |  | *psaJ_rpl33* | intergentic | LSC |
| *C.japonica* | p4 | **(AGAT)3** | 12 | 6692 | 6703 |  | *rps16_trnQ-UUG* | intergentic | LSC |
| *C.japonica* | p4 | **(GTCT)3** | 12 | 12007 | 12018 |  | *atpA* | gene | LSC |
| *C.japonica* | p4 | **(TCTT)3** | 12 | 33726 | 33737 |  | *trnT-GGU_psbD* | intergentic | LSC |
| *C.japonica* | p4 | **(TTTC)3** | 12 | 44886 | 44897 |  | *ycf3_intron* | intron | LSC |
| *C.japonica* | p4 | **(AAAT)3** | 12 | 62337 | 62348 |  | *ycf4_cemA* | intergentic | LSC |
| *C.japonica* | p4 | **(TCTA)3** | 12 | 94270 | 94281 |  | *ycf2* | gene | IRa |
| *C.japonica* | p4 | **(CCCT)3** | 12 | 109713 | 109724 |  | *rrn4.5S_rrn5S* | intergentic | IRa |
| *C.japonica* | p4 | **(GAAA)3** | 12 | 117739 | 117750 |  | *ndhD* | gene | SSC |
| *C.japonica* | p4 | **(AATA)3** | 12 | 117889 | 117900 |  | *ndhD* | gene | SSC |
| *C.japonica* | p4 | **(AAAT)3** | 12 | 120884 | 120895 |  | *ndhG_ndhI* | intergentic | SSC |
| *C.japonica* | p4 | **(AATA)3** | 12 | 122605 | 122616 |  | *ndhA_intron* | intron | SSC |
| *C.japonica* | p4 | **(GAGG)3** | 12 | 133141 | 133152 |  | *rrn5S_rrn4.5S* | intergentic | IRb |
| *C.japonica* | p4 | **(ATAG)3** | 12 | 148584 | 148595 |  | *ycf2* | gene | IRb |
| *C.azalea* | p4 | **(AGAT)3** | 12 | 6709 | 6720 |  | *rps16_trnQ-UUG* | intergentic | LSC |
| *C.azalea* | p4 | **(GTCT)3** | 12 | 12006 | 12017 |  | *atpA* | gene | LSC |
| *C.azalea* | p4 | **(TCTT)3** | 12 | 34116 | 34127 |  | *trnT-GGU_psbD* | intergentic | LSC |
| *C.azalea* | p4 | **(TTTC)3** | 12 | 45266 | 45277 |  | *ycf3_intron* | intron | LSC |
| *C.azalea* | p4 | **(AAAT)3** | 12 | 62714 | 62725 |  | *ycf4_cemA* | intergentic | LSC |
| *C.azalea* | p4 | **(TCTA)3** | 12 | 94686 | 94697 |  | *ycf2* | gene | IRa |
| *C.azalea* | p4 | **(CCCT)3** | 12 | 110110 | 110121 |  | *rrn4.5_rrn5* | intergentic | IRa |
| *C.azalea* | p4 | **(GAAA)3** | 12 | 118221 | 118232 |  | *ndhD* | gene | SSC |
| *C.azalea* | p4 | **(AATA)3** | 12 | 118371 | 118382 |  | *ndhD* | gene | SSC |
| *C.azalea* | p4 | **(AAAT)3** | 12 | 121366 | 121377 |  | *ndhG_ndhI* | intergentic | SSC |
| *C.azalea* | p4 | **(GAGG)3** | 12 | 133592 | 133603 |  | *rrn5_rrn4.5* | intergentic | IRb |
| *C.azalea* | p4 | **(ATAG)3** | 12 | 149016 | 149027 |  | *trnL-CAA_trnI-CAU* | intergentic | IRb |
| *C.crapnelliana* | p4 | **(AGAT)3** | 12 | 6705 | 6716 |  | *rps16_trnQ-UUG* | intergentic | LSC |
| *C.crapnelliana* | p4 | **(GTCT)3** | 12 | 12007 | 12018 |  | *atpA* | gene | LSC |
| *C.crapnelliana* | p4 | **(TCTT)3** | 12 | 34105 | 34116 |  | *trnM-CAU_psbD* | intergentic | LSC |
| *C.crapnelliana* | p4 | **(TTTC)3** | 12 | 45259 | 45270 |  | *ycf3_intron* | intron | LSC |
| *C.crapnelliana* | p4 | **(AAAG)3** | 12 | 46491 | 46502 |  | *ycf3_trnS-GGA* | intergentic | LSC |
| *C.crapnelliana* | p4 | **(AAAT)3** | 12 | 62700 | 62711 |  | *ycf4_cemA* | intergentic | LSC |
| *C.crapnelliana* | p4 | **(TCTA)3** | 12 | 94673 | 94684 |  | *ycf2* | gene | IRa |
| *C.crapnelliana* | p4 | **(CCCT)3** | 12 | 110116 | 110127 |  | *rrn4.5_rrn5* | intergentic | IRa |
| *C.crapnelliana* | p4 | **(GAAA)3** | 12 | 118147 | 118158 |  | *ndhD* | gene | SSC |
| *C.crapnelliana* | p4 | **(AATA)3** | 12 | 118297 | 118308 |  | *ndhD* | gene | SSC |
| *C.crapnelliana* | p4 | **(AAAT)3** | 12 | 121292 | 121303 |  | *ndhG_ndhI* | intergentic | SSC |
| *C.crapnelliana* | p4 | **(GAGG)3** | 12 | 133537 | 133548 |  | *rrn5_rrn4.5* | intergentic | IRb |
| *C.crapnelliana* | p4 | **(ATAG)3** | 12 | 148980 | 148991 |  | *ycf2* | gene | IRb |
| *C.huana* | p4 | **(AGAT)3** | 12 | 6730 | 6741 |  | *rps16_trnQ (UUG)* | intergentic | LSC |
| *C.huana* | p4 | **(GTCT)3** | 12 | 12024 | 12035 |  | *atpA* | gene | LSC |
| *C.huana* | p4 | **(TCTT)3** | 12 | 34130 | 34141 |  | *trnT (GGU)_psbD* | intergentic | LSC |
| *C.huana* | p4 | **(TTTC)3** | 12 | 45267 | 45278 |  | *ycf3_intron* | intron | LSC |
| *C.huana* | p4 | **(AAAT)3** | 12 | 62663 | 62674 |  | *ycf4_cemA* | intergentic | LSC |
| *C.huana* | p4 | **(TCTA)3** | 12 | 94624 | 94635 |  | *ycf2* | gene | IRa |
| *C.huana* | p4 | **(CCCT)3** | 12 | 110066 | 110077 |  | *rrn4.5_rrn5* | intergentic | IRa |
| *C.huana* | p4 | **(GAAA)3** | 12 | 118086 | 118097 |  | *ndhD* | gene | SSC |
| *C.huana* | p4 | **(AATA)3** | 12 | 118236 | 118247 |  | *ndhD* | gene | SSC |
| *C.huana* | p4 | **(AAAT)3** | 12 | 121231 | 121242 |  | *ndhG_ndhI* | intergentic | SSC |
| *C.huana* | p4 | **(GAGG)3** | 12 | 133446 | 133457 |  | *rrn5_rrn4.5* | intergentic | IRb |
| *C.huana* | p4 | **(ATAG)3** | 12 | 148888 | 148899 |  | *ycf2* | gene | IRb |
| *C.liberofilamenta* | p4 | **(AGAT)3** | 12 | 6742 | 6753 |  | *rps16_trnQ (UUG)* | intergentic | LSC |
| *C.liberofilamenta* | p4 | **(GTCT)3** | 12 | 12039 | 12050 |  | *atpA* | gene | LSC |
| *C.liberofilamenta* | p4 | **(TCTT)3** | 12 | 34148 | 34159 |  | *trnT (GGU)_psbD* | intergentic | LSC |
| *C.liberofilamenta* | p4 | **(TTTC)3** | 12 | 45243 | 45254 |  | *ycf3_intron* | intron | LSC |
| *C.liberofilamenta* | p4 | **(AAAT)3** | 12 | 62680 | 62691 |  | *ycf4_cemA* | intergentic | LSC |
| *C.liberofilamenta* | p4 | **(TCTA)3** | 12 | 94617 | 94628 |  | *ycf2* | gene | IRa |
| *C.liberofilamenta* | p4 | **(CCCT)3** | 12 | 110058 | 110069 |  | *rrn4.5_rrn5* | intergentic | IRa |
| *C.liberofilamenta* | p4 | **(GAAA)3** | 12 | 118066 | 118077 |  | *ndhD* | gene | SSC |
| *C.liberofilamenta* | p4 | **(AATA)3** | 12 | 118216 | 118227 |  | *ndhD* | gene | SSC |
| *C.liberofilamenta* | p4 | **(AAAT)3** | 12 | 121211 | 121222 |  | *ndhG_ndhI* | intergentic | SSC |
| *C.liberofilamenta* | p4 | **(GAGG)3** | 12 | 133427 | 133438 |  | *rrn5_rrn4.5* | intergentic | IRb |
| *C.liberofilamenta* | p4 | **(ATAG)3** | 12 | 148868 | 148879 |  | *ycf2* | gene | IRb |
| *C.luteoflora* | p4 | **(AGAT)3** | 12 | 6755 | 6766 |  | *rps16_trnQ (UUG)* | intergentic | LSC |
| *C.luteoflora* | p4 | **(GTCT)3** | 12 | 12055 | 12066 |  | *atpA* | gene | LSC |
| *C.luteoflora* | p4 | **(TCTT)3** | 12 | 34176 | 34187 |  | *trnT (GGU)_psbD* | intergentic | LSC |
| *C.luteoflora* | p4 | **(TTTC)3** | 12 | 45322 | 45333 |  | *ycf3_intron* | intron | LSC |
| *C.luteoflora* | p4 | **(AAAT)3** | 12 | 62766 | 62777 |  | *ycf4_cemA* | intergentic | LSC |
| *C.luteoflora* | p4 | **(TCTA)3** | 12 | 94757 | 94768 |  | *ycf2* | gene | IRa |
| *C.luteoflora* | p4 | **(CCCT)3** | 12 | 110199 | 110210 |  | *rrn4.5_rrn5* | intergentic | IRa |
| *C.luteoflora* | p4 | **(GAAA)3** | 12 | 118327 | 118338 |  | *ndhD* | gene | SSC |
| *C.luteoflora* | p4 | **(AATA)3** | 12 | 118477 | 118488 |  | *ndhD* | gene | SSC |
| *C.luteoflora* | p4 | **(AAAT)3** | 12 | 121472 | 121483 |  | *ndhG_ndhI* | intergentic | SSC |
| *C.luteoflora* | p4 | **(GAGG)3** | 12 | 133727 | 133738 |  | *rrn5_rrn4.5* | intergentic | IRb |
| *C.luteoflora* | p4 | **(ATAG)3** | 12 | 149169 | 149180 |  | *ycf2* | gene | IRb |
| *C.luteoflora* | p1 | **(A)10** | 10 | 32964 | 32973 |  | *trnE (UUC)_trnT (GGU)* | intergentic | LSC |
| *C.luteoflora* | p1 | **(T)14** | 14 | 33267 | 33280 |  | *trnT (GGU)_psbD* | intergentic | LSC |
| *C.luteoflora* | p1 | **(A)11** | 11 | 37663 | 37673 |  | *trnS (UGA)_psbZ* | intergentic | LSC |
| *C.luteoflora* | p1 | **(A)10** | 10 | 38301 | 38310 |  | *psbZ_trnG (GCC)* | intergentic | LSC |
| *C.luteoflora* | p1 | **(A)14** | 14 | 38532 | 38545 |  | *trnG (GCC)_tRNA-fM (CAU)* | intergentic | LSC |
| *C.luteoflora* | p1 | **(A)11** | 11 | 44213 | 44223 |  | *psaA_ycf3* | intergentic | LSC |
| *C.luteoflora* | p1 | **(A)12** | 12 | 46264 | 46275 |  | *ycf3_intron* | intron | LSC |
| *C.luteoflora* | p1 | **(A)14** | 14 | 46520 | 46533 |  | *ycf3_trnS (GGA)* | intergentic | LSC |
| *C.luteoflora* | p1 | **(A)13** | 13 | 49379 | 49391 |  | *trnT (UGU)_trnL (UAA)* | intergentic | LSC |
| *C.luteoflora* | p1 | **(T)11** | 11 | 52679 | 52689 |  | *ndhK* | gene | LSC |
| *C.luteoflora* | p1 | **(T)10** | 10 | 53266 | 53275 |  | *ndhC_trnV (UAC)* | intergentic | LSC |
| *C.luteoflora* | p1 | **(T)13** | 13 | 53418 | 53430 |  | *ndhC_trnV (UAC)* | intergentic | LSC |
| *C.luteoflora* | p1 | **(T)10** | 10 | 56379 | 56388 |  | *atpB* | gene | LSC |
| *C.luteoflora* | p1 | **(T)13** | 13 | 56813 | 56825 |  | *atpB_rbcL* | intergentic | LSC |
| *C.luteoflora* | p1 | **(T)14** | 14 | 59028 | 59041 |  | *rbcL_accD* | intergentic | LSC |
| *C.luteoflora* | p1 | **(T)14** | 14 | 60809 | 60822 |  | *accD_psaI* | intergentic | LSC |
| *C.luteoflora* | p1 | **(A)10** | 10 | 65356 | 65365 |  | *petA_psbJ* | intergentic | LSC |
| *C.luteoflora* | p1 | **(T)10** | 10 | 65494 | 65503 |  | *petA_psbJ* | intergentic | LSC |
| *C.luteoflora* | p1 | **(T)12** | 12 | 65844 | 65855 |  | *petA_psbJ* | intergentic | LSC |
| *C.luteoflora* | p1 | **(T)10** | 10 | 71045 | 71054 |  | *rps18_rpl20* | intergentic | LSC |
| *C.luteoflora* | p1 | **(T)10** | 10 | 73358 | 73367 |  | *clpP_intron* | intron | LSC |
| *C.luteoflora* | p1 | **(T)10** | 10 | 80720 | 80729 |  | *rpoA* | gene | LSC |
| *C.luteoflora* | p1 | **(T)10** | 10 | 82642 | 82651 |  | *infA_rps8* | intergentic | LSC |
| *C.luteoflora* | p1 | **(T)14** | 14 | 83167 | 83180 |  | *rps8_rpl14* | intergentic | LSC |
| *C.luteoflora* | p1 | **(A)12** | 12 | 83694 | 83705 |  | *rpl14_rpl16* | intergentic | LSC |
| *C.luteoflora* | p1 | **(T)11** | 11 | 85145 | 85155 |  | *rpl16_intron* | intron | LSC |
| *C.luteoflora* | p1 | **(T)10** | 10 | 105415 | 105424 |  | *trnI (GAU)_intron* | intron | IRa |
| *C.luteoflora* | p1 | **(A)11** | 11 | 110473 | 110483 |  | *rrn5_trnR (ACG)* | intergentic | IRa |
| *C.luteoflora* | p1 | **(T)13** | 13 | 115496 | 115508 |  | *ndhF_rpl32* | intergentic | SSC |
| *C.luteoflora* | p1 | **(T)10** | 10 | 117023 | 117032 |  | *trnL (UAG)_ccsA* | intergentic | SSC |
| *C.luteoflora* | p1 | **(A)11** | 11 | 117309 | 117319 |  | *ccsA* | gene | SSC |
| *C.luteoflora* | p1 | **(T)14** | 14 | 121508 | 121521 |  | *ndhG_ndhI* | intergentic | SSC |
| *C.luteoflora* | p1 | **(T)12** | 12 | 127487 | 127498 |  | *ycf1* | gene | SSC |
| *C.luteoflora* | p1 | **(T)12** | 12 | 129102 | 129113 |  | *ycf1* | gene | SSC |
| *C.luteoflora* | p1 | **(A)12** | 12 | 129406 | 129417 |  | *ycf1* | gene | SSC |
| *C.luteoflora* | p1 | **(A)10** | 10 | 129939 | 129948 |  | *ycf1* | gene | SSC |
| *C.luteoflora* | p1 | **(T)17** | 17 | 130320 | 130336 |  | *ycf1* | gene | SSC |
| *C.luteoflora* | p1 | **(T)11** | 11 | 133403 | 133413 |  | *trnR (ACG)_rrn5* | intergentic | IRb |
| *C.luteoflora* | p1 | **(A)10** | 10 | 138462 | 138471 |  | *trnI (GAU)_intron* | intron | IRb |
| *C.japonica* | p2 | **(AT)5** | 10 | 20461 | 20470 |  | *rpoC2* | gene | LSC |
| *C.japonica* | p2 | **(AT)5** | 10 | 83948 | 83957 |  | *rpl16_rps3* | intergentic | LSC |
| *C.japonica* | p2 | **(TA)5** | 10 | 86915 | 86924 |  | *rpl2_intron* | intron | IRa |
| *C.japonica* | p2 | **(AT)5** | 10 | 155941 | 155950 |  | *rpl2_intron* | intron | IRb |
| *C.azalea* | p2 | **(AT)5** | 10 | 20858 | 20867 |  | *rpoC2* | gene | LSC |
| *C.azalea* | p2 | **(AT)5** | 10 | 84367 | 84376 |  | *rpl16_intron* | intron | LSC |
| *C.azalea* | p2 | **(TA)5** | 10 | 87331 | 87340 |  | *rpl2_intron* | intron | IRa |
| *C.azalea* | p2 | **(AT)5** | 10 | 156373 | 156382 |  | *rpl2_intron* | intron | IRb |
| *C.crapnelliana* | p2 | **(AT)5** | 10 | 20850 | 20859 |  | *rpoC2* | gene | LSC |
| *C.crapnelliana* | p2 | **(AT)5** | 10 | 84350 | 84359 |  | *rpl16_intron* | intron | LSC |
| *C.crapnelliana* | p2 | **(TA)5** | 10 | 87318 | 87327 |  | *rpl2_intron* | intron | IRa |
| *C.crapnelliana* | p2 | **(AT)5** | 10 | 156337 | 156346 |  | *rpl2_intron* | intron | IRb |
| *C.huana* | p2 | **(AT)5** | 10 | 20869 | 20878 |  | *rpoC2* | gene | LSC |
| *C.huana* | p2 | **(AT)5** | 10 | 84283 | 84292 |  | *rpl16_intron* | intron | LSC |
| *C.huana* | p2 | **(TA)5** | 10 | 87251 | 87260 |  | *rpl2_intron* | intron | IRa |
| *C.huana* | p2 | **(AT)5** | 10 | 156263 | 156272 |  | *rpl2_intron* | intron | IRb |
| *C.liberofilamenta* | p2 | **(AT)5** | 10 | 20885 | 20894 |  | *rpoC2* | gene | LSC |
| *C.liberofilamenta* | p2 | **(AT)5** | 10 | 84294 | 84303 |  | *rpl16_intron* | intron | LSC |
| *C.liberofilamenta* | p2 | **(TA)5** | 10 | 87262 | 87271 |  | *rpl2_intron* | intron | IRa |
| *C.liberofilamenta* | p2 | **(AT)5** | 10 | 156225 | 156234 |  | *rpl2_intron* | intron | IRb |
| *C.luteoflora* | p2 | **(AT)5** | 10 | 20907 | 20916 |  | *rpoC2* | gene | LSC |
| *C.luteoflora* | p2 | **(AT)5** | 10 | 84434 | 84443 |  | *rpl16_intron* | intron | LSC |
| *C.luteoflora* | p2 | **(TA)5** | 10 | 87402 | 87411 |  | *rpl2_intron* | intron | IRa |
| *C.luteoflora* | p2 | **(AT)5** | 10 | 156526 | 156535 |  | *rpl2_intron* | intron | IRb |
| *C.japonica* | p3 | **(TTC)4** | 12 | 69730 | 69741 |  | *psaJ_rpl33* | intergentic | LSC |
| *C.azalea* | p3 | **(TTC)4** | 12 | 70124 | 70135 |  | *psaJ_rpl33* | intergentic | LSC |
| *C.crapnelliana* | p3 | **(TTC)4** | 12 | 70100 | 70111 |  | *psaJ_rpl33* | intergentic | LSC |
| *C.huana* | p3 | **(TTC)4** | 12 | 70041 | 70052 |  | *psaJ_rpl33* | intergentic | LSC |
| *C.huana* | p3 | **(ATT)4** | 12 | 74728 | 74739 |  | *clpP_psbB* | intergentic | LSC |
| *C.liberofilamenta* | p3 | **(TTC)4** | 12 | 70069 | 70080 |  | *psaJ_rpl33* | intergentic | LSC |
| *C.luteoflora* | p3 | **(TTC)4** | 12 | 70193 | 70204 |  | *psaJ_rpl33* | intergentic | LSC |
| *C.japonica* | p4 | **(AGAT)3** | 12 | 6692 | 6703 |  | *rps16_trnQ-UUG* | intergentic | LSC |
| *C.japonica* | p4 | **(GTCT)3** | 12 | 12007 | 12018 |  | *atpA* | gene | LSC |
| *C.japonica* | p4 | **(TCTT)3** | 12 | 33726 | 33737 |  | *trnT-GGU_psbD* | intergentic | LSC |
| *C.japonica* | p4 | **(TTTC)3** | 12 | 44886 | 44897 |  | *ycf3_intron* | intron | LSC |
| *C.japonica* | p4 | **(AAAT)3** | 12 | 62337 | 62348 |  | *ycf4_cemA* | intergentic | LSC |
| *C.japonica* | p4 | **(TCTA)3** | 12 | 94270 | 94281 |  | *ycf2* | gene | IRa |
| *C.japonica* | p4 | **(CCCT)3** | 12 | 109713 | 109724 |  | *rrn4.5S_rrn5S* | intergentic | IRa |
| *C.japonica* | p4 | **(GAAA)3** | 12 | 117739 | 117750 |  | *ndhD* | gene | SSC |
| *C.japonica* | p4 | **(AATA)3** | 12 | 117889 | 117900 |  | *ndhD* | gene | SSC |
| *C.japonica* | p4 | **(AAAT)3** | 12 | 120884 | 120895 |  | *ndhG_ndhI* | intergentic | SSC |
| *C.japonica* | p4 | **(AATA)3** | 12 | 122605 | 122616 |  | *ndhA_intron* | intron | SSC |
| *C.japonica* | p4 | **(GAGG)3** | 12 | 133141 | 133152 |  | *rrn5S_rrn4.5S* | intergentic | IRb |
| *C.japonica* | p4 | **(ATAG)3** | 12 | 148584 | 148595 |  | *ycf2* | gene | IRb |
| *C.azalea* | p4 | **(AGAT)3** | 12 | 6709 | 6720 |  | *rps16_trnQ-UUG* | intergentic | LSC |
| *C.azalea* | p4 | **(GTCT)3** | 12 | 12006 | 12017 |  | *atpA* | gene | LSC |
| *C.azalea* | p4 | **(TCTT)3** | 12 | 34116 | 34127 |  | *trnT-GGU_psbD* | intergentic | LSC |
| *C.azalea* | p4 | **(TTTC)3** | 12 | 45266 | 45277 |  | *ycf3_intron* | intron | LSC |
