# Supplementary material for: Complete chloroplast genome of Camellia japonica genome structures, comparative and phylogenetic analysis
Source: PLoS One. 2019 May 9;14(5):e0216645. doi: 10.1371/journal.pone.0216645 (PMC6508735; doi:10.1371/journal.pone.0216645)
Supplement: S2 Table — (DOCX) [file pone.0216645.s002.docx]

**TABLE S2 | Distribution of each SSR type in each of the six *Camellia* cp genomes.**

|  | **Category** | **SSR type** | **Number** | **Intergenic** | **Gene** | **Intron** | **LSC** | **SSC** | **IRa** | **IRb** |
| --- | --- | --- | --- | --- | --- | --- | --- | --- | --- | --- |
| *C.crapnelliana* | Mono-nucleotide | (A)10 | 3 | 1 | 1 | 1 | 1 | 1 | 0 | 1 |
|  |  | (A)11 | 8 | 7 | 1 | 0 | 7 | 1 | 0 | 0 |
|  |  | (A)12 | 6 | 4 | 1 | 1 | 4 | 1 | 1 | 0 |
|  |  | (A)13 | 1 | 1 | 0 | 0 | 1 | 0 | 0 | 0 |
|  |  | (A)14 | 2 | 2 | 0 | 0 | 2 | 0 | 0 | 0 |
|  |  | (A)17 | 1 | 1 | 0 | 0 | 1 | 0 | 0 | 0 |
|  |  | (T)10 | 13 | 9 | 3 | 1 | 10 | 2 | 1 | 0 |
|  |  | (T)11 | 2 | 0 | 1 | 1 | 2 | 0 | 0 | 0 |
|  |  | (T)12 | 7 | 4 | 3 | 0 | 3 | 3 | 0 | 1 |
|  |  | (T)13 | 4 | 4 | 0 | 0 | 4 | 0 | 0 | 0 |
|  |  | (T)14 | 4 | 3 | 0 | 1 | 3 | 1 | 0 | 0 |
|  |  | (T)15 | 1 | 1 | 0 | 0 | 1 | 0 | 0 | 0 |
|  |  | (T)17 | 2 | 1 | 1 | 0 | 1 | 1 | 0 | 0 |
|  |  | Sub-total | 54 | 38 | 11 | 5 | 40 | 10 | 2 | 2 |
|  | Di-nucleotide | (AT)5 | 3 | 0 | 1 | 2 | 2 | 0 | 0 | 1 |
|  |  | (TA)5 | 1 | 0 | 0 | 1 | 0 | 0 | 1 | 0 |
|  |  | Sub-total | 4 | 0 | 1 | 3 | 2 | 0 | 1 | 1 |
|  | Tri-nucleotide | (TTC)4 | 1 | 1 | 0 | 0 | 1 | 0 | 0 | 0 |
|  |  | Sub-total | 1 | 1 | 0 | 0 | 1 | 0 | 0 | 0 |
|  | Tetra-nucleotide | (AAAG)3 | 1 | 1 | 0 | 0 | 1 | 0 | 0 | 0 |
|  |  | (AAAT)3 | 2 | 2 | 0 | 0 | 1 | 1 | 0 | 0 |
|  |  | (AATA)3 | 1 | 0 | 1 | 0 | 0 | 1 | 0 | 0 |
|  |  | (AGAT)3 | 1 | 1 | 0 | 0 | 1 | 0 | 0 | 0 |
|  |  | (ATAG)3 | 1 | 0 | 1 | 0 | 0 | 0 | 0 | 1 |
|  |  | (CCCT)3 | 1 | 1 | 0 | 0 | 0 | 0 | 1 | 0 |
|  |  | (GAAA)3 | 1 | 0 | 1 | 0 | 0 | 1 | 0 | 0 |
|  |  | (GAGG)3 | 1 | 1 | 0 | 0 | 0 | 0 | 0 | 1 |
|  |  | (GTCT)3 | 1 | 0 | 1 | 0 | 1 | 0 | 0 | 0 |
|  |  | (TCTA)3 | 1 | 0 | 1 | 0 | 0 | 0 | 1 | 0 |
|  |  | (TCTT)3 | 1 | 1 | 0 | 0 | 1 | 0 | 0 | 0 |
|  |  | (TTTC)3 | 1 | 0 | 0 | 1 | 1 | 0 | 0 | 0 |
|  |  | Sub-total | 13 | 7 | 5 | 1 | 6 | 3 | 2 | 2 |
|  |  | Total | 72 | 46 | 17 | 9 | 49 | 13 | 5 | 5 |

**TABLE S3**（续）

|  | **Category** | **SSR type** | **Number** | **Intergenic** | **Gene** | **Intron** | **LSC** | **SSC** | **IRa** | **IRb** |
| --- | --- | --- | --- | --- | --- | --- | --- | --- | --- | --- |
| *C.luteoflora* | Mono-nucleotide | (A)10 | 7 | 4 | 1 | 2 | 5 | 1 | 0 | 1 |
|  |  | (A)11 | 5 | 4 | 1 | 0 | 3 | 1 | 1 | 0 |
|  |  | (A)12 | 3 | 1 | 1 | 1 | 2 | 1 | 0 | 0 |
|  |  | (A)13 | 1 | 1 | 0 | 0 | 1 | 0 | 0 | 0 |
|  |  | (A)14 | 3 | 3 | 0 | 0 | 3 | 0 | 0 | 0 |
|  |  | (A)15 | 1 | 1 | 0 | 0 | 1 | 0 | 0 | 0 |
|  |  | (A)16 | 1 | 1 | 0 | 0 | 1 | 0 | 0 | 0 |
|  |  | (T)10 | 10 | 5 | 3 | 2 | 8 | 1 | 1 | 0 |
|  |  | (T)11 | 5 | 2 | 2 | 1 | 4 | 0 | 0 | 1 |
|  |  | (T)12 | 6 | 4 | 2 | 0 | 4 | 2 | 0 | 0 |
|  |  | (T)13 | 3 | 3 | 0 | 0 | 2 | 1 | 0 | 0 |
|  |  | (T)14 | 5 | 5 | 0 | 0 | 4 | 1 | 0 | 0 |
|  |  | (T)17 | 1 | 0 | 1 | 0 | 0 | 1 | 0 | 0 |
|  |  | Sub-total | 51 | 34 | 11 | 6 | 38 | 9 | 2 | 2 |
|  | Di-nucleotide | (AT)5 | 3 | 0 | 1 | 2 | 2 | 0 | 0 | 1 |
|  |  | (TA)5 | 1 | 0 | 0 | 1 | 0 | 0 | 1 | 0 |
|  |  | Sub-total | 4 | 0 | 1 | 3 | 2 | 0 | 1 | 1 |
|  | Tri-nucleotide | (TTC)4 | 1 | 1 | 0 | 0 | 1 | 0 | 0 | 0 |
|  |  | Sub-total | 1 | 1 | 0 | 0 | 1 | 0 | 0 | 0 |
|  | Tetra-nucleotide | (AAAT)3 | 2 | 2 | 0 | 0 | 1 | 1 | 0 | 0 |
|  |  | (AATA)3 | 1 | 0 | 1 | 0 | 0 | 1 | 0 | 0 |
|  |  | (AGAT)3 | 1 | 1 | 0 | 0 | 1 | 0 | 0 | 0 |
|  |  | (ATAG)3 | 1 | 0 | 1 | 0 | 0 | 0 | 0 | 1 |
|  |  | (CCCT)3 | 1 | 1 | 0 | 0 | 0 | 0 | 1 | 0 |
|  |  | (GAAA)3 | 1 | 0 | 1 | 0 | 0 | 1 | 0 | 0 |
|  |  | (GAGG)3 | 1 | 1 | 0 | 0 | 0 | 0 | 0 | 1 |
|  |  | (GTCT)3 | 1 | 0 | 1 | 0 | 1 | 0 | 0 | 0 |
|  |  | (TCTA)3 | 1 | 0 | 1 | 0 | 0 | 0 | 1 | 0 |
|  |  | (TCTT)3 | 1 | 1 | 0 | 0 | 1 | 0 | 0 | 0 |
|  |  | (TTTC)3 | 1 | 0 | 0 | 1 | 1 | 0 | 0 | 0 |
|  |  | Sub-total | 12 | 6 | 5 | 1 | 5 | 3 | 2 | 2 |
|  |  | Total | 68 | 41 | 17 | 10 | 46 | 12 | 5 | 5 |

**TABLE S3**（续）

|  | **Category** | **SSR type** | **Number** | **Intergenic** | **Gene** | **Intron** | **LSC** | **SSC** | **IRa** | **IRb** |
| --- | --- | --- | --- | --- | --- | --- | --- | --- | --- | --- |
| *C.huana* | Mono-nucleotide | (A)10 | 9 | 6 | 1 | 2 | 6 | 1 | 0 | 2 |
|  |  | (A)11 | 6 | 4 | 1 | 1 | 4 | 1 | 1 | 0 |
|  |  | (A)12 | 2 | 1 | 1 | 0 | 1 | 1 | 0 | 0 |
|  |  | (A)13 | 2 | 2 | 0 | 0 | 2 | 0 | 0 | 0 |
|  |  | (A)14 | 2 | 2 | 0 | 0 | 2 | 0 | 0 | 0 |
|  |  | (A)16 | 1 | 1 | 0 | 0 | 1 | 0 | 0 | 0 |
|  |  | (T)10 | 13 | 8 | 4 | 1 | 11 | 0 | 2 | 0 |
|  |  | (T)11 | 9 | 5 | 2 | 2 | 7 | 1 | 0 | 1 |
|  |  | (T)12 | 4 | 2 | 2 | 0 | 2 | 2 | 0 | 0 |
|  |  | (T)13 | 3 | 3 | 0 | 0 | 2 | 1 | 0 | 0 |
|  |  | (T)14 | 2 | 2 | 0 | 0 | 2 | 0 | 0 | 0 |
|  |  | (T)15 | 1 | 1 | 0 | 0 | 1 | 0 | 0 | 0 |
|  |  | (T)17 | 1 | 0 | 1 | 0 | 0 | 1 | 0 | 0 |
|  |  | Sub-total | 55 | 37 | 12 | 6 | 41 | 8 | 3 | 3 |
|  | Di-nucleotide | (AT)5 | 3 | 0 | 1 | 2 | 2 | 0 | 0 | 1 |
|  |  | (TA)5 | 1 | 0 | 0 | 1 | 0 | 0 | 1 | 0 |
|  |  | Sub-total | 4 | 0 | 1 | 3 | 2 | 0 | 1 | 1 |
|  | Tri-nucleotide | (ATT)4 | 1 | 1 | 0 | 0 | 1 | 0 | 0 | 0 |
|  |  | (TTC)4 | 1 | 1 | 0 | 0 | 1 | 0 | 0 | 0 |
|  |  | Sub-total | 2 | 2 | 0 | 0 | 2 | 0 | 0 | 0 |
|  |  | (AAAT)3 | 2 | 2 | 0 | 0 | 1 | 1 | 0 | 0 |
|  |  | (AATA)3 | 1 | 0 | 1 | 0 | 0 | 1 | 0 | 0 |
|  |  | (AGAT)3 | 1 | 1 | 0 | 0 | 1 | 0 | 0 | 0 |
|  |  | (ATAG)3 | 1 | 0 | 1 | 0 | 0 | 0 | 0 | 1 |
|  |  | (CCCT)3 | 1 | 1 | 0 | 0 | 0 | 0 | 1 | 0 |
|  |  | (GAAA)3 | 1 | 0 | 1 | 0 | 0 | 1 | 0 | 0 |
|  |  | (GAGG)3 | 1 | 1 | 0 | 0 | 0 | 0 | 0 | 1 |
|  |  | (GTCT)3 | 1 | 0 | 1 | 0 | 1 | 0 | 0 | 0 |
|  |  | (TCTA)3 | 1 | 0 | 1 | 0 | 0 | 0 | 1 | 0 |
|  |  | (TCTT)3 | 1 | 1 | 0 | 0 | 1 | 0 | 0 | 0 |
|  |  | (TTTC)3 | 1 | 0 | 0 | 1 | 1 | 0 | 0 | 0 |
|  |  | Sub-total | 12 | 6 | 5 | 1 | 5 | 3 | 2 | 2 |
|  |  | Total | 73 | 45 | 18 | 10 | 50 | 11 | 6 | 6 |

**TABLE S3**（续）

|  | **Category** | **SSR type** | **Number** | **Intergenic** | **Gene** | **Intron** | **LSC** | **SSC** | **IRa** | **IRb** |
| --- | --- | --- | --- | --- | --- | --- | --- | --- | --- | --- |
| *C.liberofilamenta* | Mono-nucleotide | (A)10 | 5 | 3 | 1 | 1 | 3 | 1 | 0 | 1 |
|  |  | (A)11 | 6 | 4 | 1 | 1 | 4 | 1 | 1 | 0 |
|  |  | (A)12 | 5 | 3 | 1 | 1 | 4 | 1 | 0 | 0 |
|  |  | (A)14 | 2 | 2 | 0 | 0 | 2 | 0 | 0 | 0 |
|  |  | (A)15 | 2 | 2 | 0 | 0 | 2 | 0 | 0 | 0 |
|  |  | (T)10 | 11 | 5 | 3 | 3 | 10 | 0 | 1 | 0 |
|  |  | (T)11 | 5 | 2 | 2 | 1 | 4 | 0 | 0 | 1 |
|  |  | (T)12 | 6 | 4 | 2 | 0 | 3 | 3 | 0 | 0 |
|  |  | (T)13 | 3 | 3 | 0 | 0 | 3 | 0 | 0 | 0 |
|  |  | (T)14 | 3 | 3 | 0 | 0 | 2 | 1 | 0 | 0 |
|  |  | (T)15 | 2 | 2 | 0 | 0 | 2 | 0 | 0 | 0 |
|  |  | (T)17 | 1 | 0 | 1 | 0 | 0 | 1 | 0 | 0 |
|  |  | Sub-total | 51 | 33 | 11 | 7 | 39 | 8 | 2 | 2 |
|  | Di-nucleotide | (AT)5 | 3 | 0 | 1 | 2 | 2 | 0 | 0 | 1 |
|  |  | (TA)5 | 1 | 0 | 0 | 1 | 0 | 0 | 1 | 0 |
|  |  | Sub-total | 4 | 0 | 1 | 3 | 2 | 0 | 1 | 1 |
|  | Tri-nucleotide | (TTC)4 | 1 | 1 | 0 | 0 | 1 | 0 | 0 | 0 |
|  |  | Sub-total | 1 | 1 | 0 | 0 | 1 | 0 | 0 | 0 |
|  | Tetra-nucleotide | (AAAT)3 | 2 | 2 | 0 | 0 | 1 | 1 | 0 | 0 |
|  |  | (AATA)3 | 1 | 0 | 1 | 0 | 0 | 1 | 0 | 0 |
|  |  | (AGAT)3 | 1 | 1 | 0 | 0 | 1 | 0 | 0 | 0 |
|  |  | (ATAG)3 | 1 | 0 | 1 | 0 | 0 | 0 | 0 | 1 |
|  |  | (CCCT)3 | 1 | 1 | 0 | 0 | 0 | 0 | 1 | 0 |
|  |  | (GAAA)3 | 1 | 0 | 1 | 0 | 0 | 1 | 0 | 0 |
|  |  | (GAGG)3 | 1 | 1 | 0 | 0 | 0 | 0 | 0 | 1 |
|  |  | (GTCT)3 | 1 | 0 | 1 | 0 | 1 | 0 | 0 | 0 |
|  |  | (TCTA)3 | 1 | 0 | 1 | 0 | 0 | 0 | 1 | 0 |
|  |  | (TCTT)3 | 1 | 1 | 0 | 0 | 1 | 0 | 0 | 0 |
|  |  | (TTTC)3 | 1 | 0 | 0 | 1 | 1 | 0 | 0 | 0 |
|  |  | Sub-total | 12 | 6 | 5 | 1 | 5 | 3 | 2 | 2 |
|  |  | Total | 68 | 40 | 17 | 11 | 47 | 11 | 5 | 5 |

**TABLE S3**（续）

|  | **Category** | **SSR type** | **Number** | **Intergenic** | **Gene** | **Intron** | **LSC** | **SSC** | **IRa** | **IRb** |
| --- | --- | --- | --- | --- | --- | --- | --- | --- | --- | --- |
| *C.azalea* | Mono-nucleotide | (A)10 | 5 | 3 | 1 | 1 | 3 | 1 | 0 | 1 |
|  |  | (A)11 | 5 | 4 | 1 | 0 | 3 | 1 | 1 | 0 |
|  |  | (A)12 | 4 | 3 | 1 | 0 | 3 | 1 | 0 | 0 |
|  |  | (A)13 | 2 | 2 | 0 | 0 | 2 | 0 | 0 | 0 |
|  |  | (A)14 | 2 | 1 | 0 | 1 | 2 | 0 | 0 | 0 |
|  |  | (A)15 | 1 | 1 | 0 | 0 | 1 | 0 | 0 | 0 |
|  |  | (A)16 | 1 | 1 | 0 | 0 | 1 | 0 | 0 | 0 |
|  |  | (T)10 | 9 | 5 | 3 | 1 | 7 | 1 | 1 | 0 |
|  |  | (T)11 | 5 | 2 | 2 | 1 | 4 | 0 | 0 | 1 |
|  |  | (T)12 | 6 | 3 | 2 | 1 | 4 | 2 | 0 | 0 |
|  |  | (T)13 | 3 | 3 | 0 | 0 | 2 | 1 | 0 | 0 |
|  |  | (T)14 | 3 | 3 | 0 | 0 | 2 | 1 | 0 | 0 |
|  |  | (T)15 | 2 | 2 | 0 | 0 | 2 | 0 | 0 | 0 |
|  |  | (T)17 | 1 | 0 | 1 | 0 | 0 | 1 | 0 | 0 |
|  |  | Sub-total | 49 | 33 | 11 | 5 | 36 | 9 | 2 | 2 |
|  | Di-nucleotide | (AT)5 | 3 | 0 | 1 | 2 | 2 | 0 | 0 | 1 |
|  |  | (TA)5 | 1 | 0 | 0 | 1 | 0 | 0 | 1 | 0 |
|  |  | Sub-total | 4 | 0 | 1 | 3 | 2 | 0 | 1 | 1 |
|  | Tri-nucleotide | (TTC)4 | 1 | 1 | 0 | 0 | 1 | 0 | 0 | 0 |
|  |  | Sub-total | 1 | 1 | 0 | 0 | 1 | 0 | 0 | 0 |
|  | Tetra-nucleotide | (AAAT)3 | 2 | 2 | 0 | 0 | 1 | 1 | 0 | 0 |
|  |  | (AATA)3 | 1 | 0 | 1 | 0 | 0 | 1 | 0 | 0 |
|  |  | (AGAT)3 | 1 | 1 | 0 | 0 | 1 | 0 | 0 | 0 |
|  |  | (ATAG)3 | 1 | 1 | 0 | 0 | 0 | 0 | 0 | 1 |
|  |  | (CCCT)3 | 1 | 1 | 0 | 0 | 0 | 0 | 1 | 0 |
|  |  | (GAAA)3 | 1 | 0 | 1 | 0 | 0 | 1 | 0 | 0 |
|  |  | (GAGG)3 | 1 | 1 | 0 | 0 | 0 | 0 | 0 | 1 |
|  |  | (GTCT)3 | 1 | 0 | 1 | 0 | 1 | 0 | 0 | 0 |
|  |  | (TCTA)3 | 1 | 0 | 1 | 0 | 0 | 0 | 1 | 0 |
|  |  | (TCTT)3 | 1 | 1 | 0 | 0 | 1 | 0 | 0 | 0 |
|  |  | (TTTC)3 | 1 | 0 | 0 | 1 | 1 | 0 | 0 | 0 |
|  |  | Sub-total | 12 | 7 | 4 | 1 | 5 | 3 | 2 | 2 |
|  |  | Total | 66 | 41 | 16 | 9 | 44 | 12 | 5 | 5 |

**TABLE S3**（续）

|  | **Category** | **SSR type** | **Number** | **Intergenic** | **Gene** | **Intron** | **LSC** | **SSC** | **IRa** | **IRb** |
| --- | --- | --- | --- | --- | --- | --- | --- | --- | --- | --- |
| *C.japonica* | Mono-nucleotide | (A)10 | 3 | 2 | 1 | 0 | 2 | 1 | 0 | 0 |
|  |  | (A)11 | 10 | 8 | 1 | 1 | 7 | 1 | 1 | 1 |
|  |  | (A)12 | 6 | 4 | 1 | 1 | 5 | 1 | 0 | 0 |
|  |  | (A)14 | 1 | 1 | 0 | 0 | 1 | 0 | 0 | 0 |
|  |  | (T)10 | 10 | 6 | 4 | 0 | 10 | 0 | 0 | 0 |
|  |  | (T)11 | 5 | 2 | 2 | 1 | 2 | 1 | 1 | 1 |
|  |  | (T)12 | 7 | 4 | 2 | 1 | 5 | 2 | 0 | 0 |
|  |  | (T)13 | 4 | 4 | 0 | 0 | 3 | 1 | 0 | 0 |
|  |  | (T)15 | 2 | 2 | 0 | 0 | 1 | 1 | 0 | 0 |
|  |  | (T)17 | 1 | 0 | 1 | 0 | 0 | 1 | 0 | 0 |
|  |  | Sub-total | 49 | 33 | 12 | 4 | 36 | 9 | 2 | 2 |
|  | Di-nucleotide | (AT)5 | 3 | 1 | 1 | 1 | 2 | 0 | 0 | 1 |
|  |  | (TA)5 | 1 | 0 | 0 | 1 | 0 | 0 | 1 | 0 |
|  |  | Sub-total | 4 | 1 | 1 | 2 | 2 | 0 | 1 | 1 |
|  | Tri-nucleotide | (TTC)4 | 1 | 1 | 0 | 0 | 1 | 0 | 0 | 0 |
|  |  | Sub-total | 1 | 1 | 0 | 0 | 1 | 0 | 0 | 0 |
|  | Tetra-nucleotide | (AAAT)3 | 2 | 2 | 0 | 0 | 1 | 1 | 0 | 0 |
|  |  | (AATA)3 | 2 | 0 | 1 | 1 | 0 | 2 | 0 | 0 |
|  |  | (AGAT)3 | 1 | 1 | 0 | 0 | 1 | 0 | 0 | 0 |
|  |  | (ATAG)3 | 1 | 0 | 1 | 0 | 0 | 0 | 0 | 1 |
|  |  | (CCCT)3 | 1 | 1 | 0 | 0 | 0 | 0 | 1 | 0 |
|  |  | (GAAA)3 | 1 | 0 | 1 | 0 | 0 | 1 | 0 | 0 |
|  |  | (GAGG)3 | 1 | 1 | 0 | 0 | 0 | 0 | 0 | 1 |
|  |  | (GTCT)3 | 1 | 0 | 1 | 0 | 1 | 0 | 0 | 0 |
|  |  | (TCTA)3 | 1 | 0 | 1 | 0 | 0 | 0 | 1 | 0 |
|  |  | (TCTT)3 | 1 | 1 | 0 | 0 | 1 | 0 | 0 | 0 |
|  |  | (TTTC)3 | 1 | 0 | 0 | 1 | 1 | 0 | 0 | 0 |
|  |  | Sub-total | 13 | 6 | 5 | 2 | 5 | 4 | 2 | 2 |
|  |  | Total | 67 | 41 | 18 | 8 | 44 | 13 | 5 | 5 |
